# Advances in Avian Diagnostic Pathology: Current Trends, Challenges and Future Directions: A Review

**DOI:** 10.1002/vms3.70905

**Published:** 2026-04-03

**Authors:** Gebyaw Menge Getnet, Mengesha Ayehu Getnet, Ayenalem Shibabaw Atenaf

**Affiliations:** ^1^ Department of Veterinary Science College of Agriculture and Natural Resource Debre Markos University Debre Markos Ethiopia; ^2^ Departments of Veterinary Pathobiology College of Veterinary Medicine and Animal Science University of Gondar Gondar Ethiopia

**Keywords:** artificial intelligence, avian pathology, diagnostics, digital pathology, poultry

## Abstract

Avian pathology is the scientific study of diseases in birds, focusing on the structural, functional and molecular changes in tissues and organs caused by infections, toxins, nutritional deficiencies or others. It plays a critical role in maintaining poultry health, ensuring food security and supporting economic growth. The aim of this review is to highlight current trends, challenges and future directions in avian pathology, with a special emphasis on advancements in diagnostic approaches that enhance avian disease detection and management. However, the practical application of advanced technologies in avian pathology remains limited, particularly in Ethiopia. Recent diagnostic advancements, including immunohistochemistry, molecular techniques as well as digital pathology, have improved the detection, characterisation and management of poultry diseases. Future directions emphasise the use of artificial intelligence (AI) and machine learning for accurate diagnostics, real‐time disease monitoring and outbreak prediction. Ethiopia has achieved significant progress in avian pathology, particularly through polymerase chain reaction and histopathology. Despite ongoing advancements, the poultry industry continues to face challenges, including emerging and re‐emerging pathogens, limited access to diagnostic infrastructure, zoonotic risks and antimicrobial resistance. Therefore, strengthening biosecurity practices, promoting responsible antimicrobial use and expanding the use of molecular, digital pathology and AI‐supported diagnostic tools remain essential strategies for protecting both poultry population and public health. To further enhance disease detection and control, diagnostic capacity and professional training in avian pathology should be strengthened in Ethiopia.

## Graphical abstract

Advances in avian diagnostic pathology integrate histopathology, immunohistochemistry, molecular diagnostics and digital pathology to enhance the detection and management of poultry diseases. Emerging tools, including machine learning and artificial intelligence, further improve diagnostic accuracy, disease surveillance and outbreak prediction. The review emphasises current challenges and future directions for strengthening modern diagnostic capacity in poultry health management.

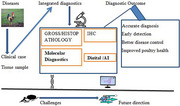

## 1 Introduction

The poultry industry occupies a central position in global food systems by supplying an abundant source of affordable, high‐quality protein in the form of meat and eggs (He et al. 2022). Ensuring poultry health and welfare is therefore critical, not only for maintaining a reliable supply of safe food but also for supporting economic development and enhancing societal wellbeing (Kaiser 2010).

In line with this importance, avian pathology, a specialised branch of veterinary science, plays a vital role in protecting poultry health by studying diseases that affect both domestic poultry and wild bird populations (Ishtiaq 2021). Poultry diseases have considerable economic and social impacts, with their occurrence influenced by multiple factors such as geo‐climatic conditions, population density, management practices and immunisation status, leading to high morbidity and mortality in chickens, increased medication and veterinary costs and substantial losses in both production and market value (Rahman and Adhikary 2016; Asfaw et al. 2021a, 2021b).

Moreover, poultry health and productivity are further compromised by infectious agents, toxic substances and nutritional imbalances, with some avian diseases, such as avian influenza, having particularly severe effects (Team, R.C. 2023). The detection of highly virulent strains often requires regulatory measures, including restrictions on poultry trade, which can adversely impact industry profitability (Chaves Hernández 2014). The movement of people and animals can facilitate the introduction of diseases into poultry facilities (Savelieff et al. 2018; Salles et al. 2023).

In avian pathology, the diagnostic process has progressively shifted from the traditional veterinary approach, which focused on individual birds, to a comprehensive assessment of the health of entire flocks (Shwetha et al. 2024). On‐farm, diagnostic activities comprise routine sampling and investigations in line with health control programmes (Team, R.C. 2023). In the field, diagnostic procedures are initiated as soon as flock health is compromised, with morbidity and/or mortality serving as primary indicators. Investigations begin with the compilation of a comprehensive case history encompassing relevant flock characteristics, management practices and infection or disease factors (Liebhart et al. 2023).

Recent technological advancements, including molecular techniques, immunohistochemistry (IHC) and digital pathology, have increasingly been applied to enhance the detection, characterisation and monitoring of avian diseases (Neethirajan 2022). These approaches enable detailed investigations of host–pathogen interactions and tissue‐level changes, facilitating early and accurate disease diagnosis (Mbelwa et al. 2021). It is evident that artificial intelligence (AI) and machine learning (ML) are likely to play an integral role in the future of disease diagnostics, both in field and laboratory settings (Guarino et al. 2017).

Therefore, avian pathology is recognised as a crucial approach for diagnosing various poultry diseases. Despite these advancements, several challenges remain. The application of molecular, immunohistochemical and digital pathology diagnostic techniques is limited. Comparative analyses of conventional versus modern diagnostic methods are scarce, and the integration of AI/ML into routine avian diagnostics remains underexplored. This review addresses these gaps by summarising recent technological advancements, evaluating their practical applicability and highlighting future directions for improving avian disease diagnosis and management.

## 2 Current Trends and Diagnostic Advances

Veterinary pathology is the branch of pathology concerned with the investigation of disease and disease processes in non‐human species, and it is a comprehensive field within veterinary medicine that focuses on diagnosing diseases in animals, including birds, forming the foundation for avian pathology (Brown 2008). Avian pathology is the scientific discipline that studies diseases in avian species, including both domestic poultry and wild bird populations (Kozdruń et al. 2015; Nemeth et al. 2016). According to these authors, avian pathology is essential for understanding and controlling diseases in both domestic poultry and wild bird populations.

**FIGURE 1:**
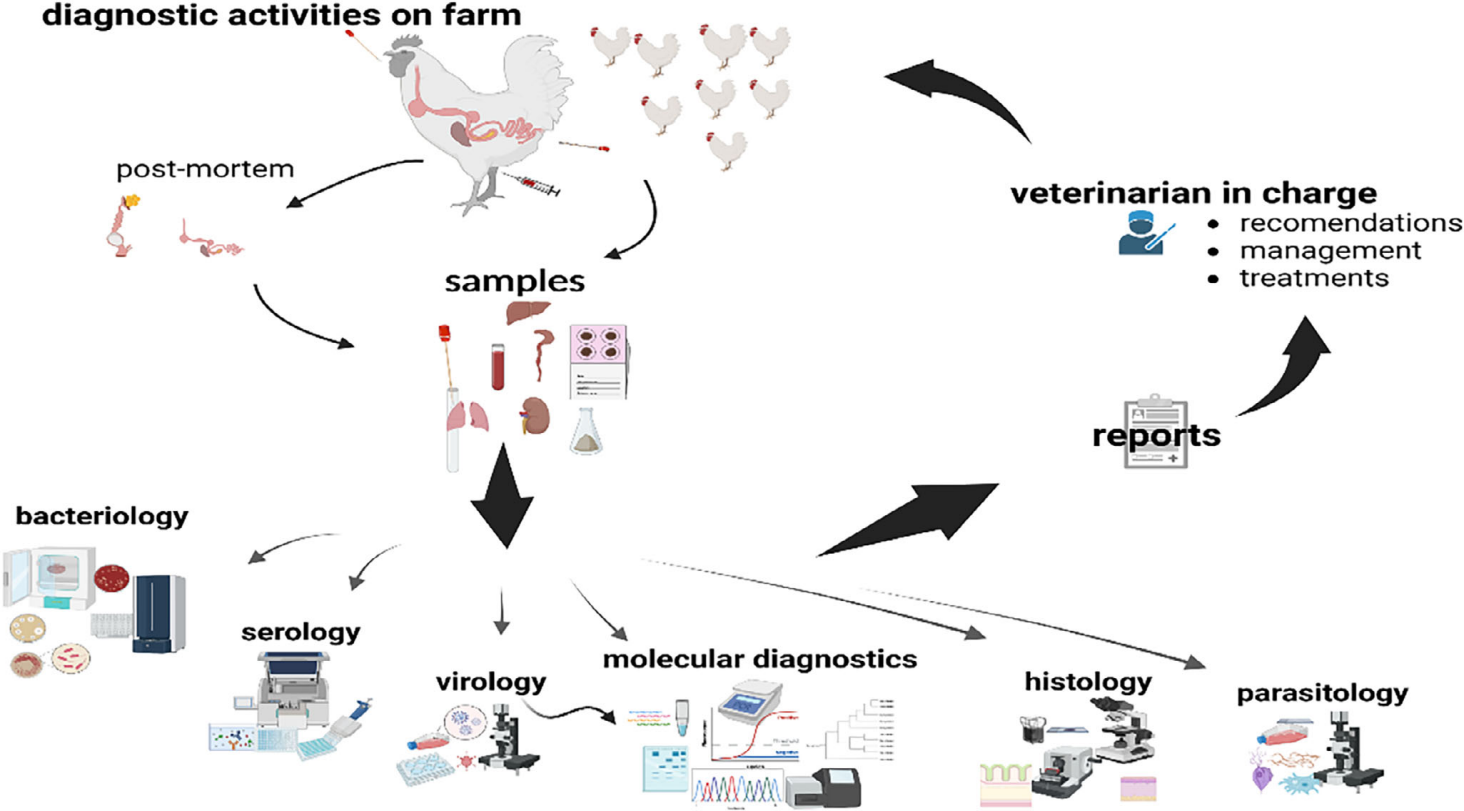
Diagram demonstrating the diagnosis of infectious poultry diseases (Liebhart et al. 2023).

Traditionally, avian disease diagnosis has relied on clinical observations, gross pathology, histopathology, microbiological culture and serological techniques (Figure 1) (Liebhart et al. 2023). These approaches have provided valuable diagnostic information, but they exhibit notable limitations in sensitivity, specificity and efficiency (Ishtiaq 2021). For instance, overlapping clinical signs and histopathological lesions among different avian diseases can result in misdiagnosis or delayed identification, which may compromise timely intervention (Raji and Aliyu 2025). Moreover, the emergence of novel bacterial and viral pathogens, as well as diseases with unidentified aetiologies, presents additional diagnostic challenges in avian pathology (Liebhart et al. 2023).

Recent advances in IHC, digital pathology and molecular diagnostics have transformed avian pathology by enhancing specificity, sensitivity and diagnostic speed (Fatima et al. 2024; Raji and Aliyu 2025). These advancements collectively strengthen the accuracy and efficiency of avian disease diagnosis and surveillance.

### 2.1 Gross Pathology

**FIGURE 2:**
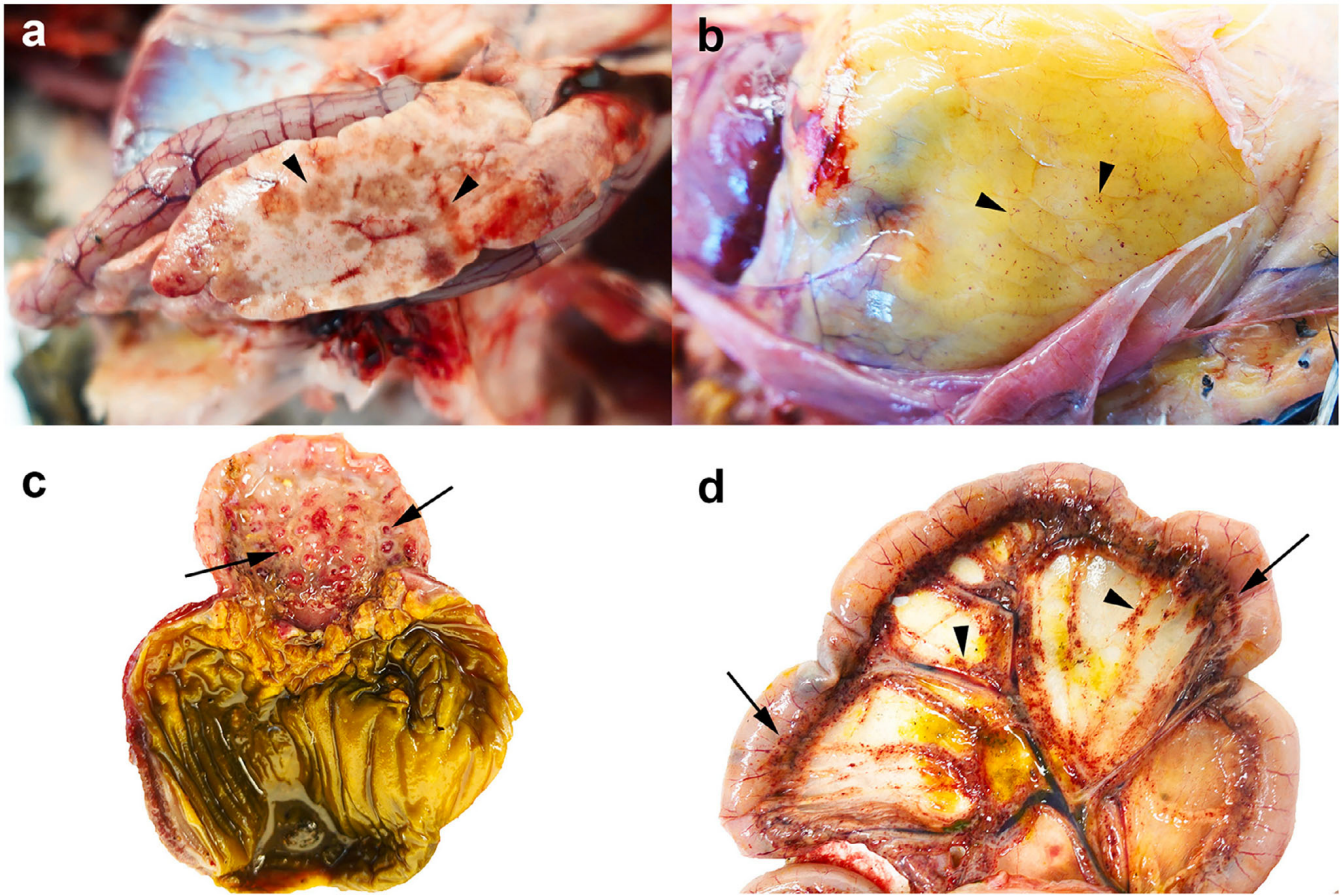
Gross lesions in birds infected with highly pathogenic avian influenza virus (H5N1). Observed lesions include pancreatic necrosis, commonly multifocal to coalescing tan discolouration and occasionally haemorrhagic (arrowheads; duck, a); petechial haemorrhages on the coelomic fat (arrowheads; chicken, b); haemorrhage of the proventricular mucosa, predominantly localised around the proventricular ducts (arrow; chicken, c); and haemorrhages on the intestinal serosa (arrow, mesenteric border) as well as within the mesentery (arrowheads; chicken, d) (Lean, Vitores, et al. 2022).

Traditionally, avian pathology has relied on morphological assessment, encompassing both macroscopic features visible to the naked eye and microscopic details revealed through histological examination (Araki 2017). Gross pathology remains a vital pillar of diagnostic practice, providing essential information for disease diagnosis, prognosis and therapeutic decision‐making through the systematic evaluation of tissues at the macroscopic level (Figure 2) (Kumar et al. 2021; Lester 2010; Hoda and Patel 2018). According to these authors, gross pathology continues to play a fundamental role in guiding accurate diagnosis, prognosis and effective therapeutic decision‐making in avian disease investigation.

### 2.2 Histopathology

**FIGURE 3:**
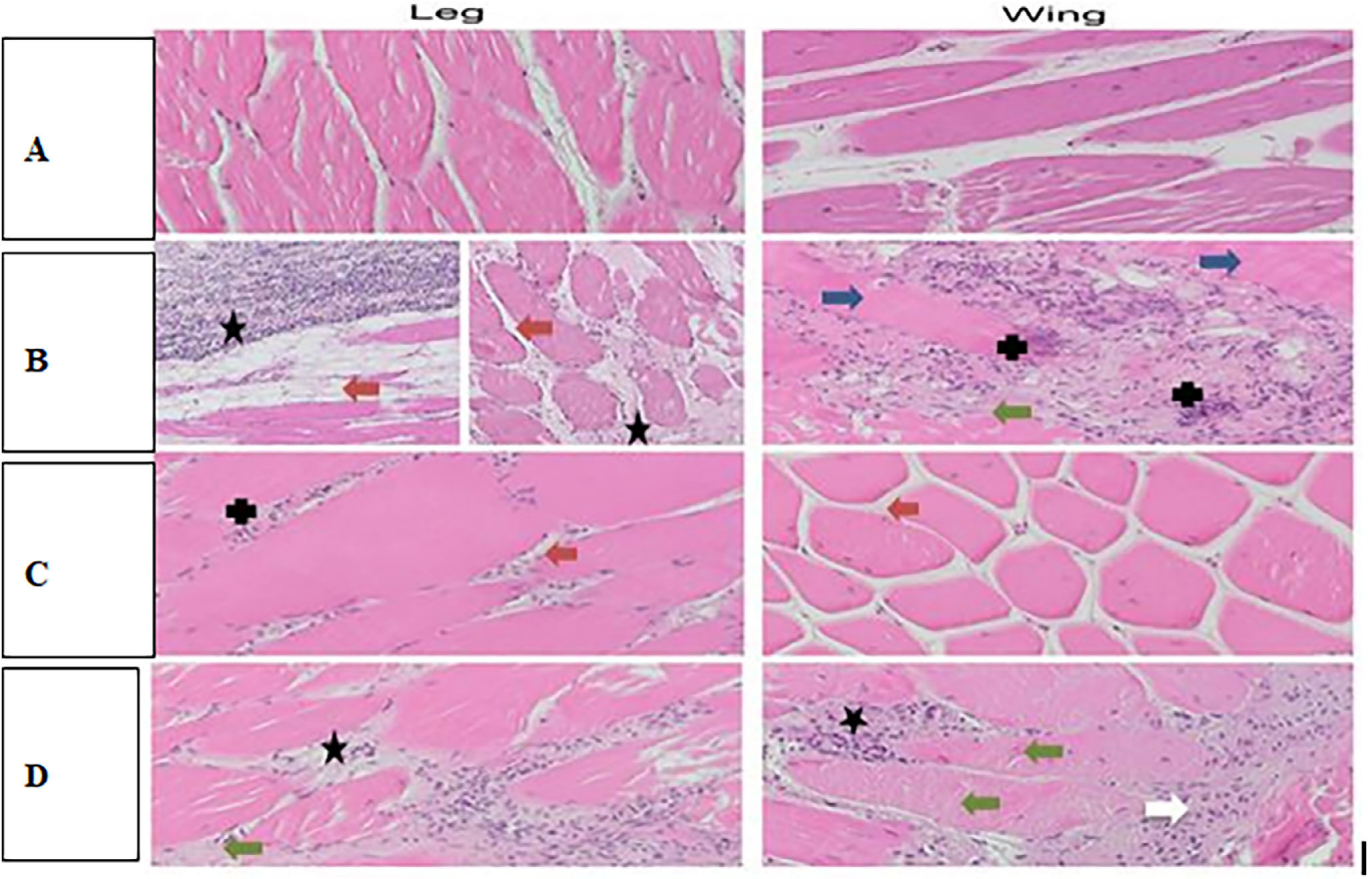
Histopathological sections of chicken legs and wings: Sections were stained with haematoxylin and eosin (H&E) and examined under a magnification of 400×, except for B‐leg samples, which were viewed at 200×. In sample a, no pathological changes (NCh) were observed in either the leg or wing tissues. In sample b, the leg tissue exhibited fibrosis (star) and increased intercellular space (red arrow), whereas the wing tissue was characterised by haemorrhage (green arrow), necrosis (blue arrow) and granulocytic infiltration (+ sign). In sample c, the leg tissue showed mild granulocytic infiltration (+ sign), whereas the wing tissue displayed increased intercellular space (red arrow). In sample d, fibrosis (star) and haemorrhage (green arrow) were evident in the leg tissue, whereas the wing tissue demonstrated fibrosis (star), mild haemorrhage (green arrow) and mononuclear infiltration (white arrow) (Libera et al. 2024).

**FIGURE 4:**
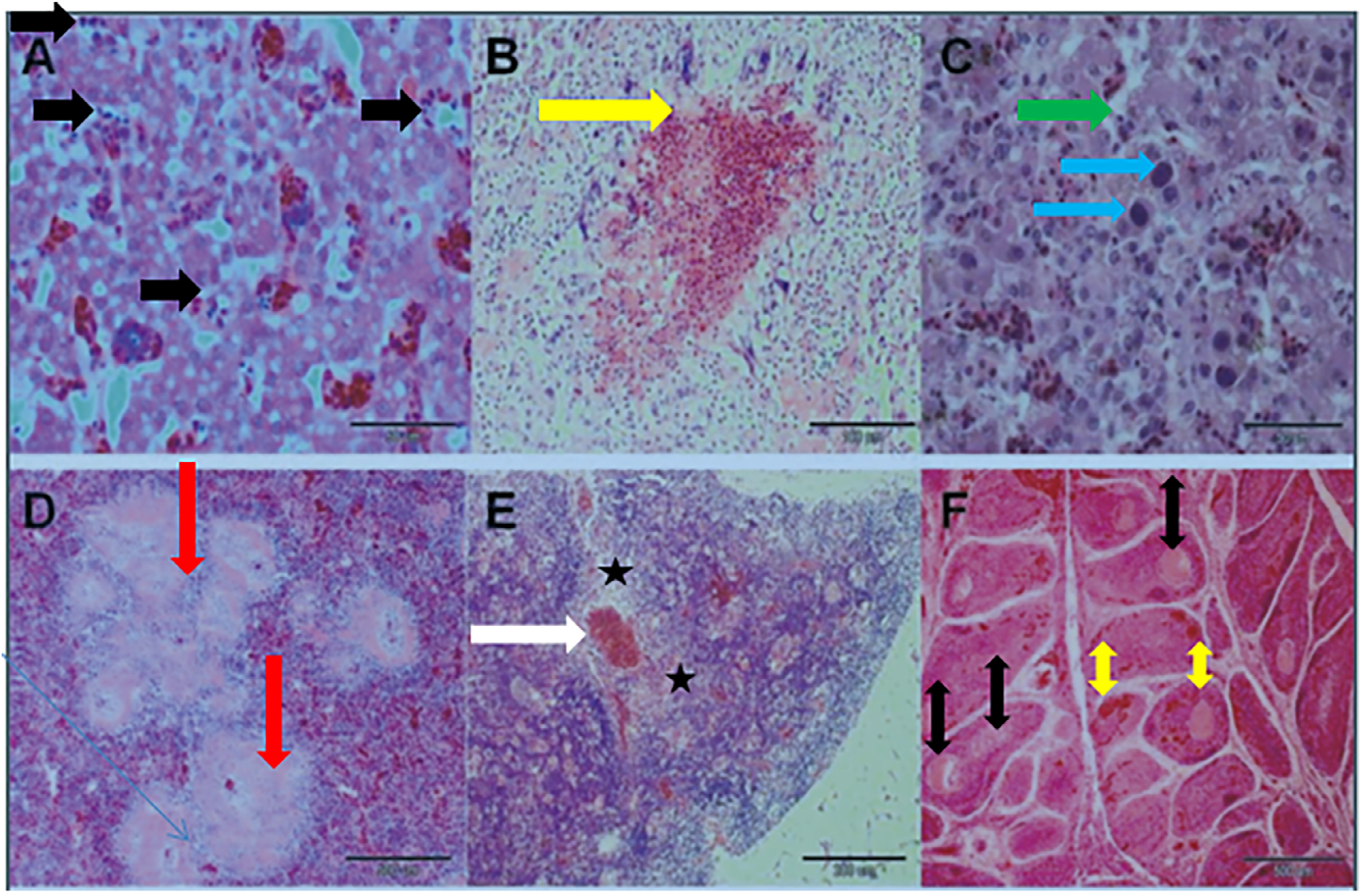
Histopathological lesions in multiple organs: (a) liver: fatty degeneration of hepatocytes with bacterial emboli (black arrow), 400×. (b) Liver: necrotic areas surrounded by multinucleated giant cells (yellow arrow), 200×. (c) Liver: coagulative necrosis (green arrow) and large basophilic intranuclear inclusion bodies in hepatocytes (blue arrow), compatible with inclusion body hepatitis (IBH), 400×. (d) Spleen: coagulative necrosis with lymphocyte depletion (red arrow), 40×. (e) Thymus: reduced lymphocyte density, medullary necrosis (black star) and hyperaemia (white arrow), 100×. (f) Bursa of Fabricius: lymphocyte depletion, necrosis (double black arrow) and follicular haemorrhage with interstitial oedema (double yellow arrow), characteristic of infectious bursal disease (IBD), 40× (Dolka et al. 2012).

**FIGURE 5:**
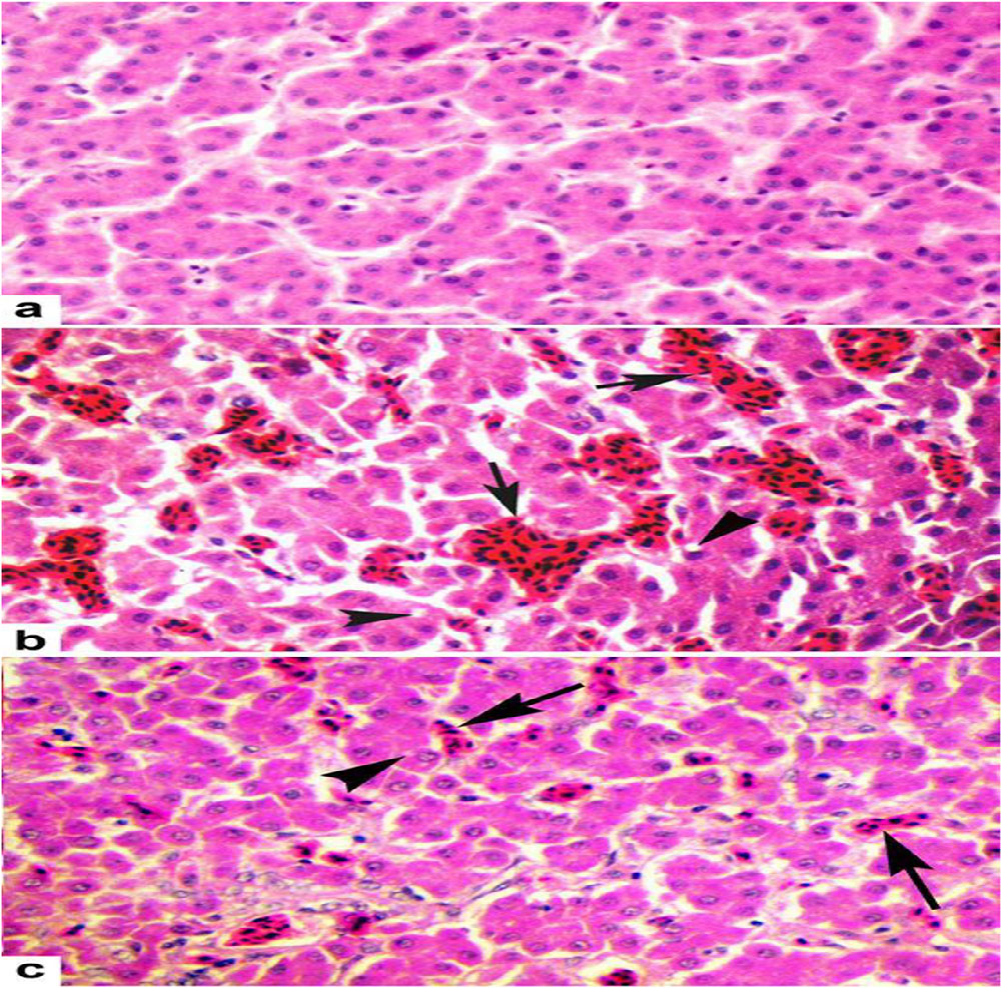
Histopathology of the liver: (a) control birds showing normal hepatic architecture; (b) arsenic (As)‐treated broiler chickens showing severe vascular congestion (arrows) and cytoplasmic vacuolation (arrowheads); and (c) arsenic plus vitamin C (As + Vit C)‐treated birds showing mild congestion and cytoplasmic vacuolation. H&E staining, ×40 (Khan et al. 2013).

Examination of tissue samples is regarded as the gold standard for disease diagnosis and is critical for assessing tissue pathology across all animal species, including birds (Fadhail 2025). Histopathological analysis allows the evaluation of host organ alterations at both the organ and cellular levels, with various tissue preparations employed to elucidate structural and pathological changes (Figures 3, 4, 5) (Chaudhari et al. 2023). Typically, tissue specimens are initially fixed in formalin to preserve architecture and prevent autolysis (Nojima et al. 2017). Subsequently, the samples are dehydrated and embedded in paraffin to provide structural support for thin sectioning. Sections are then stained using haematoxylin and eosin (H&E), which delineates nuclei and cytoplasmic components, thereby facilitating clear visualisation of cellular and tissue morphology (Liebhart et al. 2023).

In H&E staining, haematoxylin specifically stains the nuclei, whereas eosin counterstains the cytoplasm and various extracellular components (Makki 2016; Lean, Núñez, et al. 2022). Histopathological examination of avian tissues is typically conducted following standardised protocols to ensure consistency, accuracy and reproducibility of findings (Fadhail 2025).

Advances in digital pathology have further enhanced histological analysis, particularly through whole‐slide imaging (WSI), which enables detailed tissue evaluation using tools for measuring tumour margins, quantifying cell proliferation and analysing tissue architecture (Pantanowitz et al. 2011). Nevertheless, the potential of digital histopathology remains limited by variability in tissue preparation and staining protocols, which can affect image quality and analytical outcomes. To address these challenges, novel approaches such as three‐dimensional histological reconstructions and multiplex staining techniques are being developed to provide more comprehensive insights into tissue morphology (Fatima et al. 2024).

### 2.3 Avian Clinical Pathology

Avian clinical pathology is a critical tool in the diagnosis and management of avian diseases (Baral 2025). In avian clinical pathology, the complete blood cell count (CBC) serves as a fundamental diagnostic tool for assessing the health status of birds (Sakas 2002). It provides critical insights into haematological alterations associated with infectious, inflammatory or metabolic diseases and has been demonstrated to be one of the most sensitive indicators for detecting subclinical or early stage illnesses in avian patients (Bhattacherjee et al. 2021).

To ensure reliable results and to minimise artefacts, blood collection should be performed promptly following the initiation of manual restraint and using proper venipuncture techniques (Piccione and Hokamp 2020). In chickens and turkeys, the brachial (wing) vein is the most commonly used site for blood collection (Guzman et al. 2008).

The use of anticoagulant is essential for accurate haematologic evaluation in avian species. The most widely used anticoagulants are EDTA and lithium heparin, which are suitable for avian CBCs, as they preserve cellular morphology, reduce artefacts, maintain sample stability over time and prevent thrombocyte aggregation during blood smear evaluation (Guzman et al. 2008; Briscoe et al. 2010; Piccione and Hokamp 2020). According to these authors, anticoagulants are crucial for ensuring accurate and reliable haematological assessment in avian species.

#### 2.3.1 Avian Erythrocytes

Unlike mammalian red blood cells, avian erythrocytes are nucleated, which poses analytical challenges in haematological assessments (Beaufrère and Vergneau‐Grosset 2021). As a result, manual techniques remain widely employed in avian haematology due to the limited availability of automated analysers specifically optimised for birds (Jones 1999; Mitchell and Johns 2008).

The assessment of the avian red blood cell system relies on several key parameters, including packed cell volume (PCV), total erythrocyte count, haemoglobin concentration and erythrocyte morphology (Thrall et al. 2012). Among these indices, total erythrocyte concentration, PCV and haemoglobin levels are influenced by several intrinsic factors, such as age, sex and hormonal status (Jones 1999; Mitchell and Johns 2008). In most avian species, the normal PCV ranges from approximately 35% to 55%, and avian erythrocytes have a shorter life span, typically between 25 and 45 days depending on the species (Hebert et al. 1989; Thrall et al. 2012).

**FIGURE 6:**
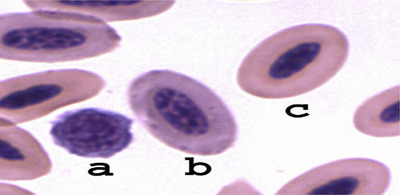
Stages of avian erythrocyte maturation: immature erythrocyte with round shape, open chromatin and basophilic cytoplasm (a); polychromatophilic erythrocyte with basophilic cytoplasm and elliptic shape (b); mature erythrocyte with elliptic shape, centrally located purple nucleus and orange cytoplasm (c) (Mitchell and Johns 2008).

Erythrocyte morphology can be assessed in the monolayer region of a blood smear by evaluating cell size (anisocytosis), shape abnormalities (poikilocytosis, such as elliptocytes or spherocytes) and haemoglobinisation defects (hypochromia or hyperchromia), providing information on erythropoiesis and overall red blood cell health. However, a mild degree of polychromasia and slight anisocytosis is commonly observed in healthy birds (Figure 6) (Jones 1999; T. W. Campbell and Ellis 2007a, 2007b). According to these authors, evaluating erythrocyte morphology provides insights into red blood cell health, with mild polychromasia and slight anisocytosis being normal in healthy birds.

#### 2.3.2 Avian Leukocytes Evaluation

The evaluation of avian leukocytes typically includes a total leukocyte count, differential leukocyte count and a morphologic assessment of the leukocytes (Briscoe et al. 2010). The leukogram provides a detailed overview of the types and numbers of leukocytes circulating in the blood, allowing for the assessment of immune status and the detection of abnormalities (Beaufrère and Vergneau‐Grosset 2021). This information is useful for identifying inflammation or infection, serving as a prognostic indicator of disease and detecting specific conditions that directly affect leukocytes, such as leukaemia or haemoparasitic infections (Jones 1999).

**FIGURE 7:**
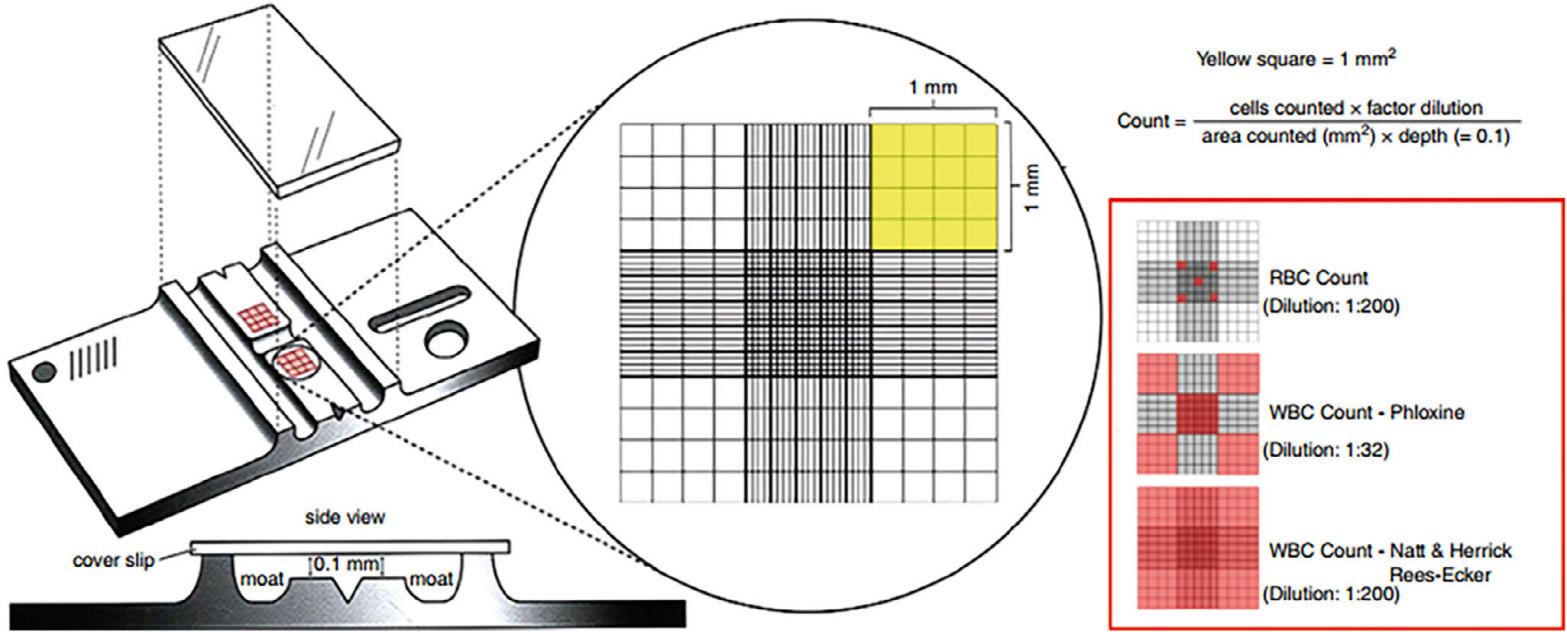
Haemocytometer and a detailed view of the counting chambers: The red insert box shows the counting areas for both white blood cell (WBC) and red blood cell counts, depending on the technique and the dilution factor. The general formula for haemocytometer‐based cell count is also provided above (Beaufrère and Vergneau‐Grosset 2021).

The determination of white blood cell (WBC) counts in birds is commonly performed using haemocytometers in combination with various staining techniques. This procedure is typically conducted manually through microscopic examination and visual assessment by a trained observer. Because all avian blood cells are nucleated, automated analysers designed for mammalian blood cannot reliably distinguish leukocytes from erythrocytes and thrombocytes (Figure 7) (Briscoe et al. 2010; Campbell et al. 2015; Raji and Aliyu 2025).

**FIGURE 8:**
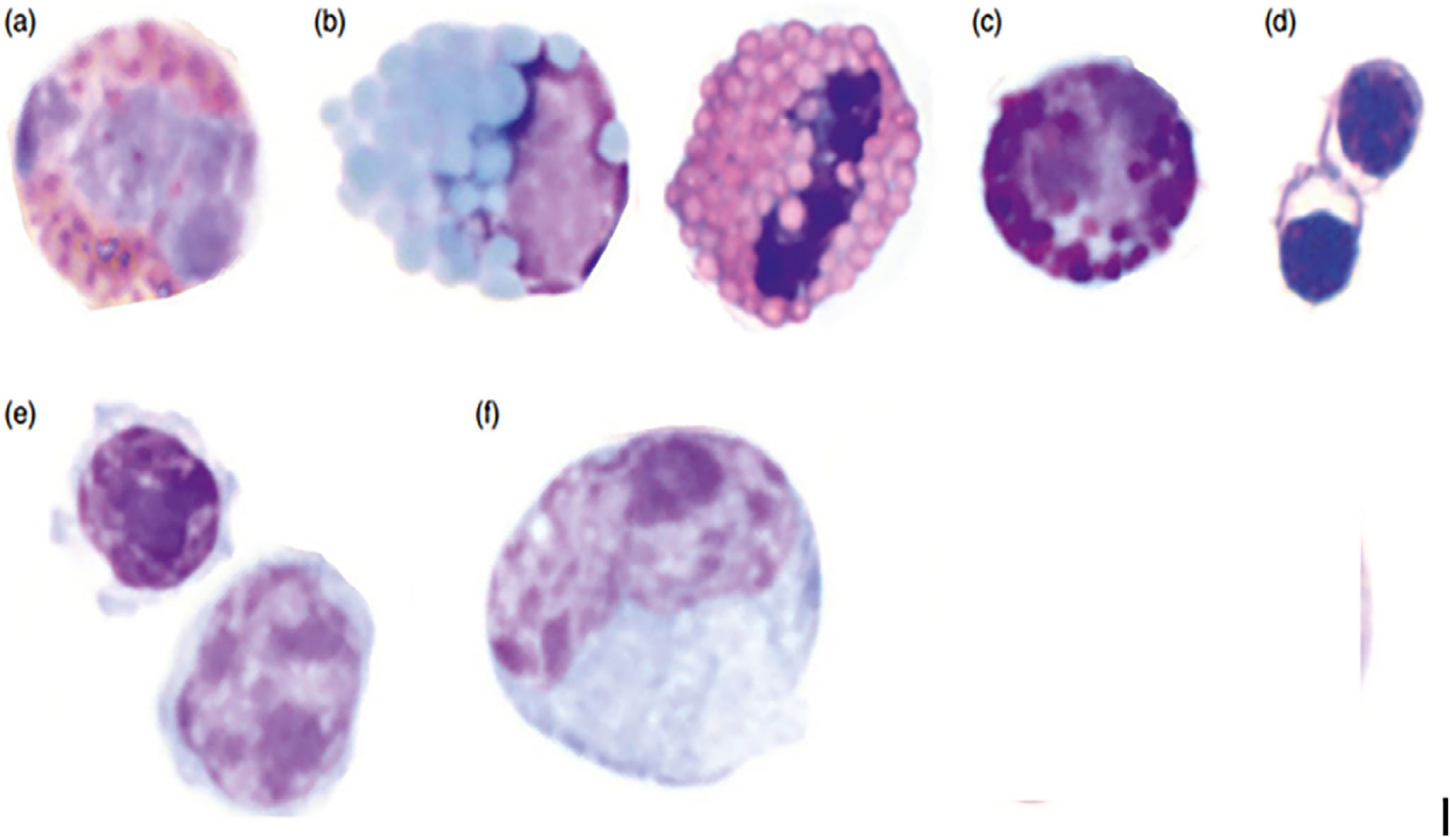
Morphology of avian white blood cells in wright‐stained smears: (a) heterophil; (b) eosinophils (blue in parrot, red in raptors); (c) basophil; (d) thrombocytes; (e) small and large lymphocytes; and (f) monocyte (Beaufrère and Vergneau‐Grosset 2021).

The total leukocyte mass, or leukon, comprises the granulocytes (heterophils, eosinophils and basophils), the mononuclear cells (lymphocytes and monocytes) and thrombocytes, as shown in Figure 8 (Jones 1999; Campbell and Grant 2022).

#### 2.3.3 Urinalysis

In avian species, urine and faeces are excreted together through the cloaca, which makes the collection of pure urine samples difficult (Harris 2004). Avian urine is generally collected from a voided sample by removal of cage paper and thorough cleaning of the cage surface (Murray 2003). A needle and syringe or capillary tube can be used to aspirate urine and minimise faecal contamination (Murray 2003). Although ureteral catheterisation has been described, this technique requires anaesthesia and is technically challenging to perform (Harr 2002). Urine can be separated from the rest of the droppings by utilising the capillary action of a glass capillary tube. When the tube is gently placed in contact with the droppings, the liquid portion is naturally drawn into the tube, allowing the urine to be collected separately from the solid faecal material and urates (Harris 2004). This method provides a simple and non‐invasive way to obtain urine with minimal faecal contamination, thereby improving the quality of the sample for diagnostic evaluation (Murray 2003).

The most valuable component of avian urinalysis is the examination of urine sediment. In some cases, the presence of granular or hyaline casts may represent the only clinicopathological evidence of renal disease (Harris 2004). In avian urine, most uric acid is present as a white to light yellow colloidal suspension known as urates. These urates consist of small, spherical aggregates with diameters ranging from 0.5 to 15 µm and are composed of uric acid, sodium and/or potassium salts and protein (Harr 2002; Beaufrère and Vergneau‐Grosset 2021).

Normal avian urine consists of a clear fluid component, and the volume of urine varies among species, reflecting adaptations to different diets and environmental conditions. In most clinically healthy birds, urine specific gravity ranges from 1.005 to 1.020, and the urine is generally acidic. The normal urine sediment typically contains uric acid precipitates and crystals, along with sloughed squamous epithelial cells (Harr 2002).

### 2.4 Immunohistochemistry

Histopathology remains a cornerstone of disease diagnosis and the study of tissue alterations through microscopic examination; however, diagnostic challenges often arise due to overlapping histomorphological features among different diseases and cell types (Levenson et al. 2013). To overcome these limitations, IHC has emerged as a powerful complementary tool, offering enhanced specificity and sensitivity in the detection of cellular markers and pathogen‐associated antigens (Mebratie and Dagnaw 2024). This technology has now expanded into broader clinical and research applications, including diagnosis, prognosis, therapeutic decision‐making and the study of disease pathogenesis, bridging the gap between traditional histopathology and molecular pathology (Leong and Leong 2006).

**FIGURE 9:**
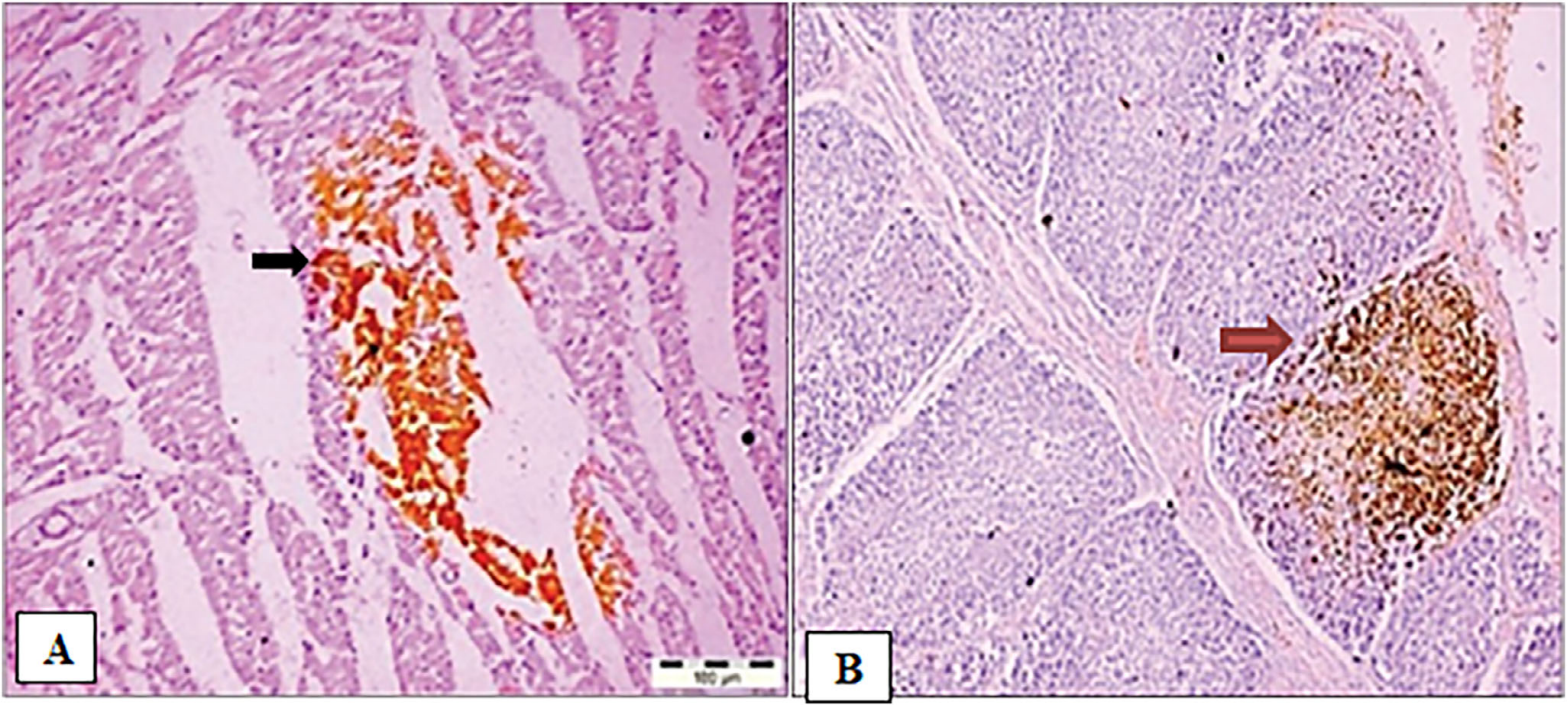
Immunohistochemical detection: (a) immunohistochemical detection of infectious bursal disease virus (IBDV) antigen in the cardiac muscle fibres (black arrow), ×20. (b) Bursal tissue section showing focally extensive localisation of IBDV antigen (red arrow), immunohistochemistry, ×40 (Singh et al. 2015).

IHC integrates three core disciplines: immunology, histology and chemistry. The fundamental concept is the demonstration of antigens within tissue sections (Figure 9) by means of specific antibodies (Ramos‐Vara 2005). Immunoglobulin molecules possess antigen‐binding sites that enable precise interaction with target antigens (Nakazawa et al. 2016). Antigen–antibody binding is demonstrated with a coloured histochemical reaction visible by light or fluorescent microscopy. Antibodies can be either monoclonal or polyclonal, and they react with various epitopes on the antigen (Ramos‐Vara and Miller 2014; Mebratie and Dagnaw 2024). Overall, monoclonal and polyclonal antibodies (pAbs) allow specific visualisation of antigens through coloured histochemical reactions.

During routine histopathological processing, formalin fixation and paraffin embedding preserve tissue structure but frequently mask or modify antigenic sites, preventing effective antibody binding (Hecke 2002). Antigen retrieval methods were introduced to overcome this challenge by reversing fixation‐induced changes and restoring antigenicity. Two main approaches are commonly used: heat‐induced antigen retrieval (HIER), which employs heat in buffered solutions, and enzymatic digestion, which utilises proteolytic enzymes to unmask hidden epitopes (Murata et al. 2019). These techniques dramatically increase the range of antigens detectable in routinely processed tissues, particularly in formalin‐fixed, paraffin‐embedded (FFPE) samples. As a result, antigen retrieval expanded the diagnostic, prognostic and research applications of IHC, firmly establishing the method as an essential tool in modern pathology (Ramos‐Vara 2005).

The IHC process can be divided into three phases. Phase 1, pre‐analytical, begins with sample collection and includes essential preparatory steps such as tissue fixation, trimming, embedding and sectioning using a microtome (Ramos‐Vara and Miller 2014). Phase 2, analytical, begins with deparaffinisation of tissue sections, followed by pre‐incubation steps such as antigen retrieval and blocking of nonspecific binding sites, and continues with incubation of the primary antibody, application of detection systems to visualise the antigen–antibody complex, and finally counterstaining and cover‐slipping of the slides (Stills 2012). Phase 3, post‐analytical, involves the interpretation of staining results, preparation of the IHC report and verification of results through evaluation of positive and negative controls (Taylor 2006).

The antigen–antibody reaction in IHC is not naturally visible under the microscope and therefore requires labelling for detection. The most widely used labels are enzymes, particularly peroxidase and alkaline phosphatase. These enzymes act on specific substrates in the presence of chromogens to produce a coloured precipitate (Hecke 2002). For instance, previous studies have reported the presence of infectious bursal disease virus (IBDV) antigen in the bursal interfollicular connective tissue, bursal epithelium and cardiac muscle fibres. The distribution of the antigen has been observed to vary considerably, ranging from localised red or brown deposits to focal aggregations and, in some cases, diffuse patterns (Singh et al. 2015).

#### 2.4.1 Monoclonal and pAbs

**FIGURE 10:**
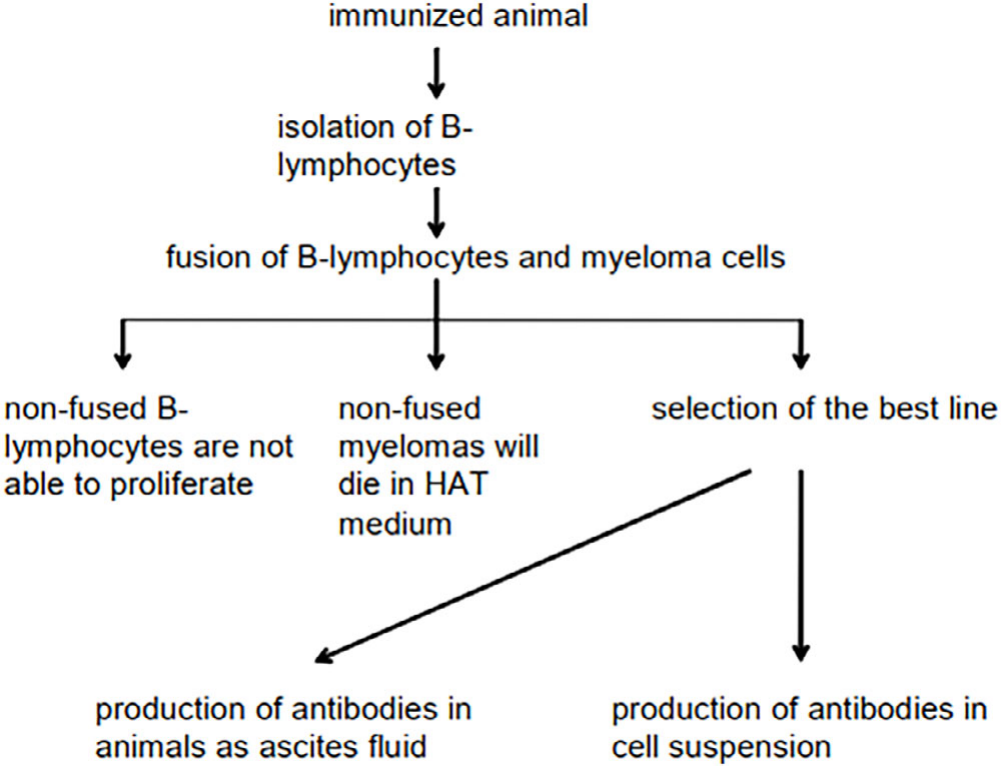
Scheme of monoclonal antibody production (Pohanka 2009).

Monoclonal antibodies (mAbs) are predominantly produced in mice. The process begins with the injection of mice with a purified immunogen (antigen, Ag) (Pohanka 2009). Following the induction of an immune response, B lymphocytes (antibody‐producing cells) are harvested from the spleen (Korsnes Ervik 2016). As isolated B cells have a limited lifespan, they are fused with immortal mouse myeloma cells to generate hybridomas. Subsequent selection of hybridomas with the desired specificity yields hybrid cells capable of continuous proliferation. These hybridomas secrete immunoglobulins, which target a single epitope and tend to be more specific (Figure 10) (Ramos‐Vara 2005; Ramos‐Vara and Borst 2016; Magaki et al. 2018). These studies collectively demonstrate that monoclonal antibody production via hybridoma technology enables the sustained generation of highly specific immunoglobulins, supporting their widespread application in IHC and diagnostic avian pathology.

**FIGURE 11:**
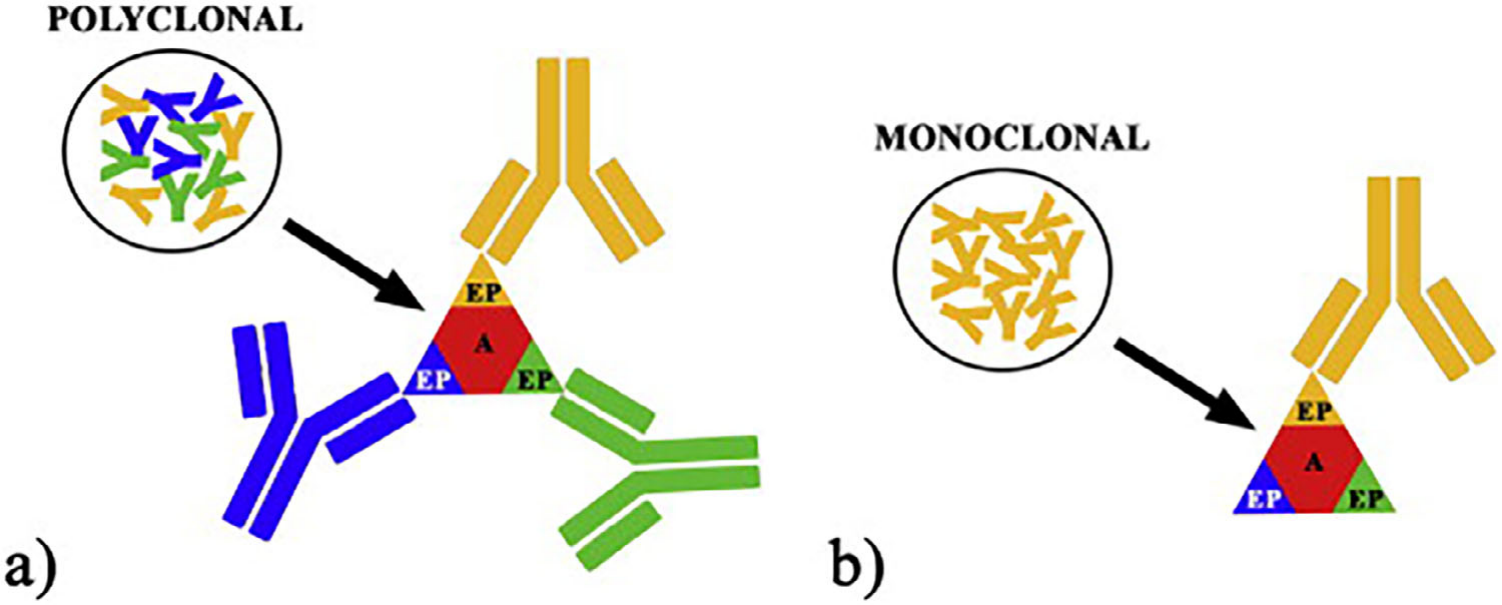
Schematic representation of polyclonal and monoclonal antibodies: (a) polyclonal antibodies recognise multiple epitopes (EP) on a single antigen (a), whereas (b) monoclonal antibodies bind to a single epitope on the antigen (Vázquez‐Gutiérrez and Langton 2015).

The term pAbsy originally referred to the total population of antibodies present in the serum of an animal. These antibodies are produced by multiple B‐cell clones that differentiate into plasma cells following stimulation by an immunogen (Nakazawa et al. 2016). In contrast, pAbs are typically generated in various animal species, including rabbits, horses, goats and chickens, and they exhibit broader reactivity and higher affinity but lower specificity. The production involves immunising animals with a purified molecule containing the antigen of interest (Stills 2012). The resulting humoral immune response leads to the generation of diverse plasma cell clones, each producing antibodies against different epitopes of the immunogen. Antibodies are then harvested by collecting serum rich in immunoglobulins. This polyclonal nature enables recognition of multiple epitopes, offering advantages in detecting heterogeneous antigens, as shown in Figure 11 (Kabiraj et al. 2015; Magaki et al. 2018). Overall, the polyclonal nature of these antibodies enables recognition of multiple epitopes, enhancing their utility in detecting heterogeneous antigens and supporting diverse applications in research and diagnostics.

#### 2.4.2 Direct and Indirect Detection Methods of IHC

Direct detection is a one‐step method in which the primary antibody is directly conjugated to a reporter molecule, such as biotin, fluorochromes or enzymes (Bunea and Zarnescu 2001). This approach is especially useful for antigens that are highly expressed or in situations where secondary antibodies can cause unwanted background staining due to the tissue type or the species of the primary antibody (Kabiraj et al. 2015). For example, in mouse lymph node staining, using labelled primary mouse antibodies is preferred because secondary antibodies against mouse immunoglobulins can also bind to the natural immunoglobulins present in the lymph nodes, leading to strong nonspecific staining. In such cases, direct detection with primary antibodies conjugated to a fluorophore or enzyme is the better option (Kalyuzhny 2016). Direct detection offers several advantages, including the elimination of the secondary antibody incubation step, which makes the method faster and easier to perform (Childs 2014). However, this approach has limitations, such as reduced sensitivity for many antigens in routinely processed tissues, and each primary antibody must be individually conjugated with fluorophores or enzymes, which significantly increases the overall cost (Taylor et al. 2013).

**FIGURE 12:**
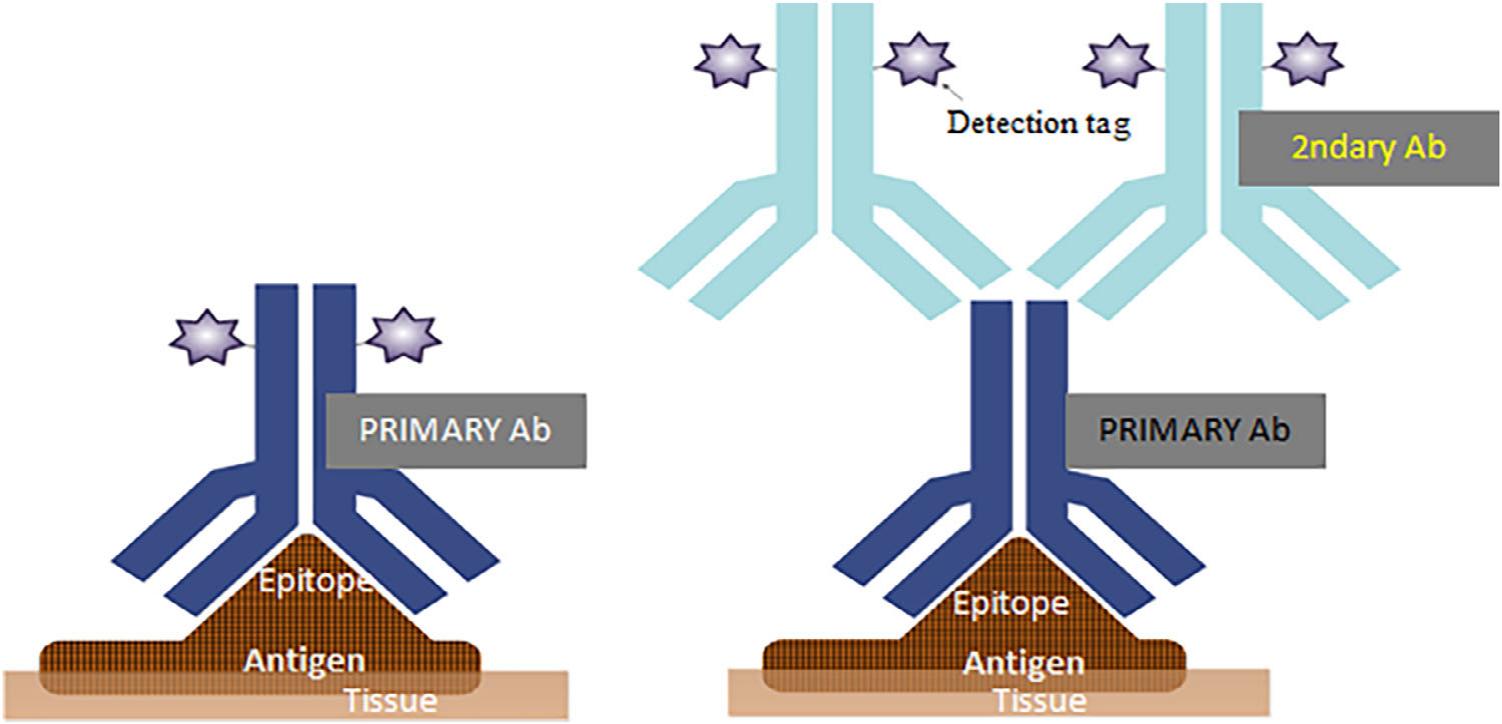
Immunohistochemical detection of tissue antigens: direct detection of a target antigen (left) and indirect detection (right) (Murata et al. 2019).

The need for more sensitive detection systems for antigens with low expression levels led to the development of two‐step, or indirect, detection methods. In this approach, an unlabelled primary antibody is applied first, followed by a labelled secondary antibody that is raised against the primary antibody and conjugated to fluorophores or enzymes (Shojaeian et al. 2018; Murata et al. 2019). Moreover, in indirect methods, the primary antibodies retain their full avidity because they remain unlabelled, allowing multiple labelled secondary antibodies to bind to each primary antibody (Figure 12) (Murata et al. 2019). This not only enhances reaction intensity but also enables the detection of lower amounts of antigen using smaller quantities of primary antibody. Indirect methods are also more practical than direct methods, as the same labelled secondary antibody can be used to detect different primary antibodies raised in the same species (Ramos‐Vara 2004). Despite the advantages described above, indirect immunostaining methods have certain limitations, including the use of secondary antibodies, which require additional controls and blocking steps. Due to this, nonspecific staining may occur when the secondary antibody binds to unintended tissue targets. In such cases, blocking reagents must be applied to the tissue sections, which can be time‐consuming and increase the overall cost of the IHC experiment (Shojaeian et al. 2018).

In IHC, digital pathology has improved the standardisation of marker quantification, particularly for well‐established markers such as Ki‐67 (Torlakovic et al. 2024). Nevertheless, the current implementation of IHC software remains in development. Although software‐based analysis has been validated for certain markers, many other IHC markers still lack sufficient validation for routine clinical use (Pantanowitz et al. 2011). Furthermore, existing algorithms often encounter difficulties with variations in staining intensity and background noise, resulting in inconsistent outcomes. Future advancements may focus on integrating AI to enhance the accuracy of quantitative IHC analysis and on developing universal protocols that standardise both staining and imaging processes across laboratories (Fatima et al. 2024).

### 2.5 Molecular Diagnostics

The diagnostic methods used in avian pathology, such as clinical sign observation, gross and histopathology, microbiology and serological techniques, have been valuable tools for disease diagnosis (Raji and Aliyu 2025). However, these methods have limitations in terms of sensitivity, specificity and time consumption and can result in misdiagnosis or delayed diagnosis due to similarities in clinical signs and lesions exhibited in many avian diseases (Raji and Omar 2022). The limitations of traditional diagnostic methods have underscored the critical need for the emergence of molecular diagnostics as a revolutionary force in avian pathology (Liu et al. 2023).

Recent advances in molecular biology and sequencing technologies are providing significant insights into the diversity of viruses as well as the dynamic changes in host and viral gene expression, allowing a better understanding of the molecular pathogenesis of diseases (Nair 2018). Moreover, the incorporation of molecular diagnostics in avian pathology has resulted in the creation of convenient and user‐friendly platforms for on‐site testing (Liebhart et al. 2023).

In avian pathology, molecular diagnostics are utilised to detect and analyse pathogen‐specific DNA and RNA molecules, although successful detection indicates only the presence of the pathogen's nucleic acid (Liebhart et al. 2023). With each passing year, an increasing number of laboratories use nucleic acid technology to detect and differentiate pathogens of avian species (Wannaratana et al. 2021). Many of these approaches involve the polymerase chain reaction (PCR), or reverse transcriptase (RT)‐PCR when the target nucleic acid is RNA, to amplify selected parts of the pathogen's genome (Cavanagh 2001).

PCR and real‐time PCR have become standard for detecting pathogens, including Newcastle disease virus, infectious bronchitis virus and *Mycoplasma* species (Nair 2018). Nucleic acids (RNA or DNA) of pathogens of interest can be directly detected in a sample by several PCR‐based tests, followed by sequencing analysis to specifically identify the microorganisms involved (Bustin and Jellinger 2023). Phylogenetic trees elaborated using sequencing data can provide useful information on the relationships of different organisms based on the percentage of similarity (Crispo 2024).

Viral genome amplification using the RT‐PCR method for diagnosing poultry diseases is common; it can detect only a single virus at a time. The multiplex PCR technique was subsequently developed to detect more than one virus simultaneously (Banakar et al. 2016). Quantitative real‐time polymerase chain reaction (rt‐qPCR) is commonly used for viral detection. This technique uses fluorescent molecular or chemical dyes coupled with molecular oligonucleotide probes to stain the PCR products (Raza et al. 2025).

#### 2.5.1 Loop‐Mediated Isothermal Amplification (LAMP)

Nucleic acid amplification, particularly PCR‐based methods, has significantly improved diagnostic accuracy in both humans and animals, including birds (Zelník 2021). However, PCR has limitations, as it depends on high‐precision thermal cyclers, a stable power supply and complex detection systems, which restrict its application in resource‐limited settings (Crispo 2024).

To overcome these challenges, LAMP was developed in 2000 as a highly efficient technique capable of amplifying low DNA copy numbers under isothermal conditions (Enyetornye et al. 2025). LAMP is a widely applied modification of PCR that eliminates the requirement for an expensive thermal cycler, thereby facilitating its use under field conditions (Table 1) (Naidu 2019). Advantages of LAMP include (1) no requirement for a thermal cycler, as it can be performed using a simple water bath, (2) no need for post‐amplification steps for result visualisation, (3) rapid results, typically within 1 h, (4) higher sensitivity compared to conventional PCR and (5) tolerance to inhibitory substances (Padzil et al. 2021). However, limitations include (1) the target sequence must be less than 300 bp, (2) higher risk of carry‐over contamination and (3) the requirement for a high strand‐displacement enzyme (Balachandran 2025).

**TABLE 1:** Comparison of loop‐mediated isothermal amplification (LAMP) and polymerase chain reaction (PCR) assays.

Feature	PCR	LAMP
**Amplification**	Thermal cycling amplification of RNA or DNA	Isothermal amplification of RNA or DNA
**Sensitivity**	High	High and comparable to PCR
**Specificity**	High with proper primer design	High when primers are properly optimised
**Time**	2–3 h	30–60 min
**Prone to presence of irrelevant DNA**	More prone	Less prone
**Equipment and cost**	High cost due to the need for a thermal cycler and consistent reagent supply	Low cost, requiring minimal equipment
**Method of detection**	Gel electrophoresis or fluorescence	Colour change, turbidity or fluorescence
**Suitability for field conditions**	Primarily used in laboratory settings	Suitable for field or on‐site diagnostic use

*Source*: Enyetornye et al. (2025).

In general, the LAMP method is characterised by the use of at least four primers that specifically recognise six regions of the target DNA sequence (Padzil et al. 2021). The main primers include a forward outer primer (F3) and a backward outer primer (B3), in addition to two inner primers: the forward inner primer (FIP) and the backward inner primer (BIP), which generate stem‐loop structures crucial for amplification, and other optional loop primers, the loop forward (LF) and loop backward (LB), which further accelerate the amplification process by providing additional priming sites (Tsai et al. 2012). Moreover, the inclusion of swarm primers targeting upstream regions of the FIP and BIP binding sites can further reduce reaction time (Table 2) (Padzil et al. 2021).

**TABLE 2:** Role of primers in the loop‐mediated isothermal amplification (LAMP) assay.

Primer type	Function
F3 and B3 (outer)	Initiate amplification
FIP and BIP (inner)	Form stem‐loop structures, drive amplification
LF and LB (loop)	Accelerate DNA synthesis by providing extra priming sites
Swarm primers	Shorten amplification time by targeting upstream regions

Abbreviations: B3, backward outer primer; BIP, backward inner primer; F3, forward outer primer; FIP, forward inner primer; LB, loop backward; LF, loop forward (Notomi et al. 2000).

**FIGURE 13:**
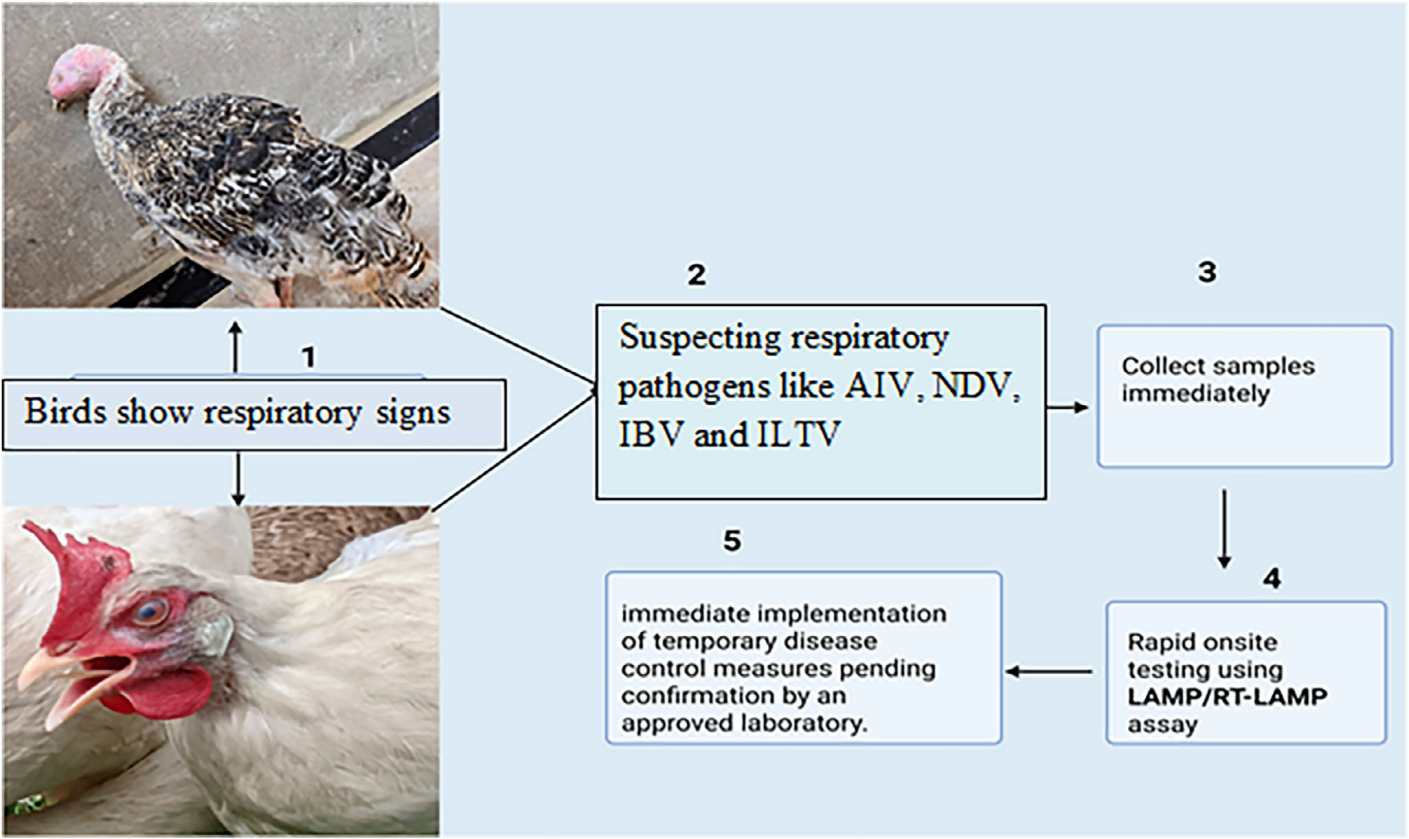
Workflow of the LAMP assay for the diagnosis of poultry respiratory viruses (Enyetornye et al. 2025). LAMP, loop‐mediated isothermal amplification.

A critical factor underlying the development of LAMP is the unique function of Bst DNA polymerase, an enzyme characterised by strong strand‐displacement activity that enables efficient DNA amplification under isothermal conditions (Notomi et al. 2000; Ma et al. 2016). Bst DNA polymerase, isolated from *Geobacillus stearothermophilus*, exhibits strong strand‐displacement and helicase‐like activity, enabling it to unwind double‐stranded DNA without the need for thermal denaturation. Unlike conventional polymerases, Bst functions efficiently at a constant temperature of 60–70°C and is not hindered by secondary DNA structures (Tsai et al. 2012). These properties make it an ideal platform for developing isothermal screening systems, particularly in diagnostic applications, where molecular detection can be performed rapidly, cost‐effectively, and with high portability across diverse settings (Figure 13) (Padzil et al. 2021).

#### 2.5.2 Next‐Generation Sequencing (NGS)

NGS has transformed the field of genomics by providing unprecedented insights into genome structure, function and dynamics (Fu et al. 2023). This innovative technology has facilitated extensive research, enabling scientists to investigate the complexities of genetic information with remarkable depth and precision (Goodwin et al. 2016).

In avian pathology, NGS has substantially advanced the understanding of avian microbiomes by enabling comprehensive analysis of genetic material from entire microbial communities within avian samples (Afonso and Afonso 2023). This technology allows rapid sequencing of large amounts of DNA and RNA, facilitating precise identification and characterisation of diverse avian pathogens, including those with large or complex genomes (Satam et al. 2023). Particularly, random NGS represents a powerful tool for obtaining comprehensive analyses of nucleic acids directly from diagnostic samples, facilitating the detection and characterisation of complex or mixed infections with greater efficiency than traditional diagnostic methods (Raza et al. 2025; Nafea et al. 2023). This capacity not only enhances the speed and accuracy of pathogen identification but also contributes to more effective prevention, surveillance and control strategies in poultry disease management (Cibulski et al. 2021; Afonso and Afonso 2023). NGS offers a comprehensive and efficient approach for the detection and characterisation of avian pathogens, thereby enhancing diagnostic accuracy and informing effective poultry disease management strategies.

**FIGURE 14:**
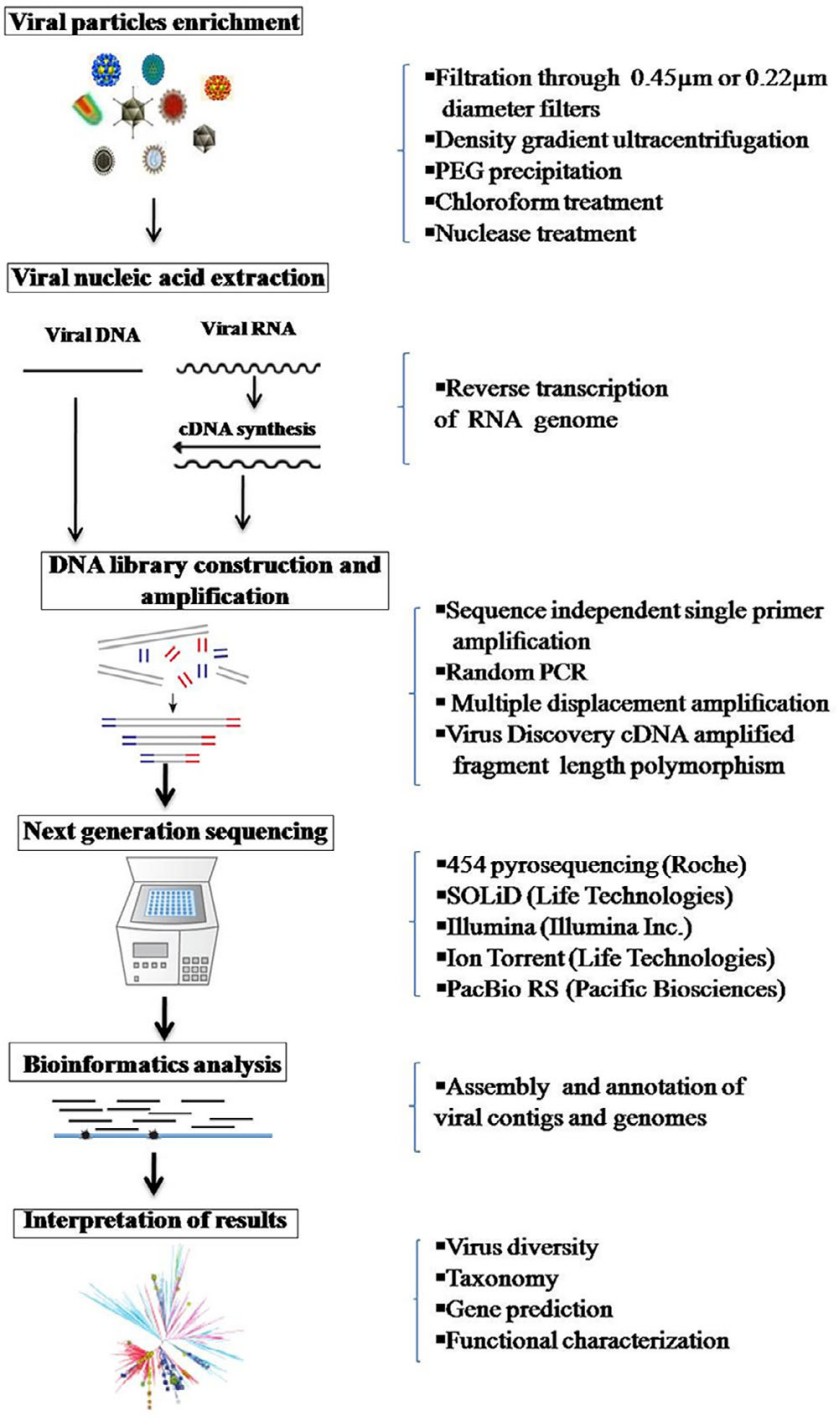
Workflow of next‐generation sequencing (NGS) for virus detection: The process follows a systematic workflow (Kapgate et al. 2015). PCR, polymerase chain reaction.

A range of commercially available high‐throughput sequencing platforms are currently in use, each differing in sequencing principles, speed, cost and read length (Raza et al. 2025). Despite these variations, the fundamental workflow of NGS remains consistent and typically involves four key steps: sample preparation, sequence‐independent amplification, high‐throughput sequencing and subsequent bioinformatics analysis, as illustrated in Figure 14 (Kapgate et al. 2015).

### 2.6 Digital Pathology

**FIGURE 15:**
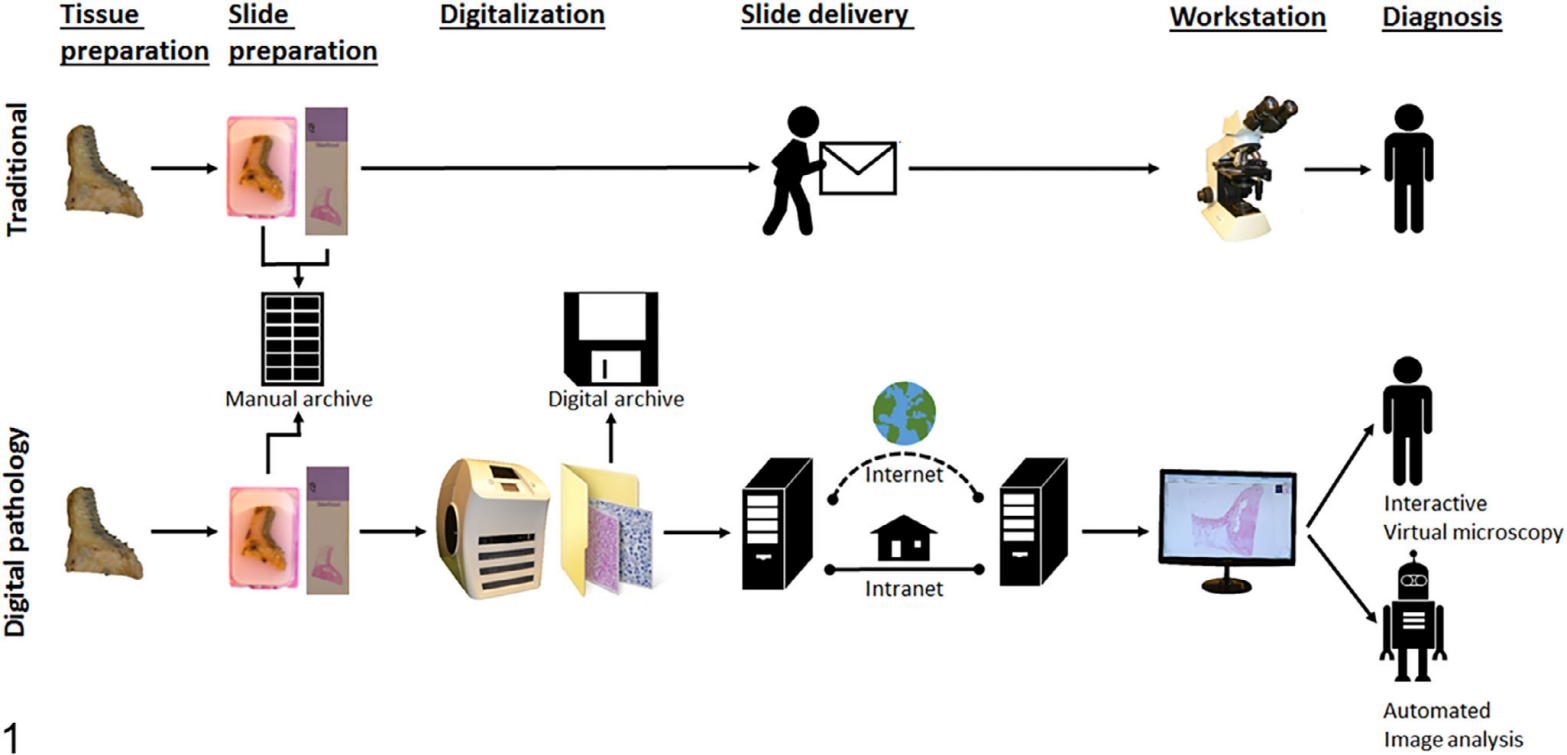
Comparison of digital pathology and traditional workflow: Digital pathology, distinguished from conventional pathology, involves the digitisation of histological sections using a slide scanner to generate whole‐slide images. These digital images can subsequently be managed and reviewed on a computerised workstation by a pathologist through interactive virtual microscopy (Bertram and Klopfleisch 2017; Ancheta et al. 2024).

Digital pathology is a transformative approach in pathology that involves the digitisation of pathology information, including slides and data (Brown 2008). The transition from traditional microscopy to digital platforms has enabled new opportunities in tissue analysis, data sharing and remote diagnostics (Figure 15) (He et al. 2012).

The main concept related to digital pathology is WSI, or scanning a histopathological slide in order to digitise it (Pantanowitz et al. 2018). After scanning, a digitised histopathological slide is a collection of digital images assembled into a single digital file (Brown 2008). A digitised histopathological slide can be viewed on a screen using specialised software, allowing the entire slide to be reviewed both at low power and under high magnification (Kiehl 2022; Mezei et al. 2024). Veterinary histopathological diagnostic services across the world have adopted this technology, mainly for case review and the digitisation of histopathology educational resources (Fadhail 2025).

There are four key processes within WSI scanners: (1) image acquisition, involving hardware that can capture and display images, usually a high‐resolution trinocular microscope camera with controls for illumination intensity, objective lenses and a mechanical stage with coarse and fine focusing mechanisms; (2) processing, in which WSIs are sequentially captured as a series of individual image tiles or lines, combined to resemble a replica of the original static slide; (3) storage, in which scanned images are saved digitally; and (4) visualisation via a computer workstation (Tang et al. 2021; Ancheta et al. 2024).

**FIGURE 16:**
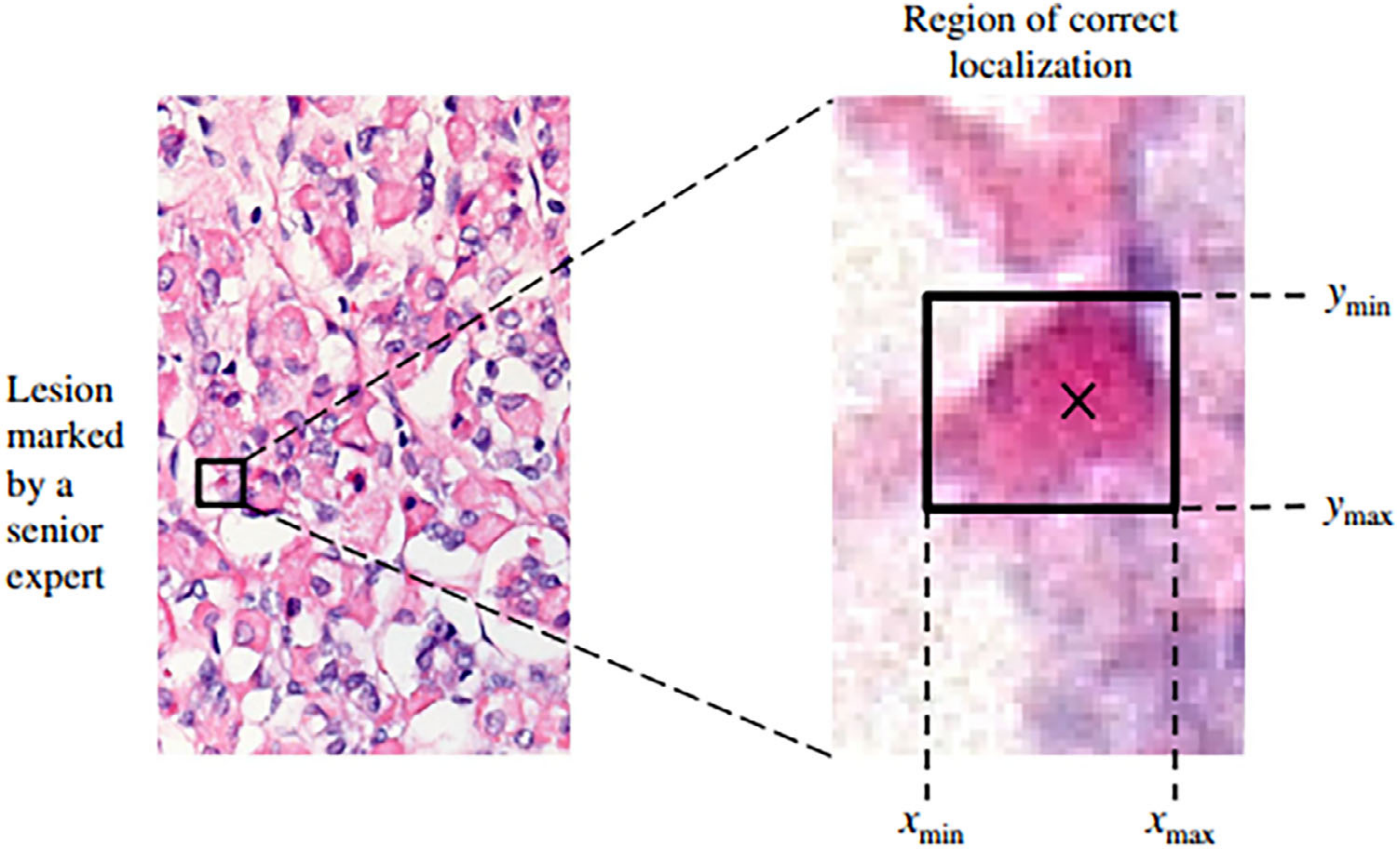
Location‐level mark classification: A mark is considered a true positive (TP) if it falls within the acceptance region surrounding the actual lesion; otherwise, it is classified as a false positive (FP). The acceptance region is defined as a manually delineated rectangular area, determined by the maximum width (*x*_max − *x*_min) and height (*y*_max − *y*_min) of the corresponding lesion in the reference image. Actual lesions are those annotated by the senior expert with a confidence rating exceeding 60% (Platiša et al. 2017).

In general, the focus has been on examining several factors that affect image acquisition, management and display within WSI systems. These factors include blurring, which may result from thick or folded tissue, incorrect focus or vibrations during scanning; colour and gamma parameters, which are typically influenced by display system settings but can also vary due to slide staining or differences in scanning devices; and noise, which tends to be lower in live tissue and higher in dead tissue samples (Platiša et al. 2017). In digital pathology, reference images are often derived from high‐resolution source images through selective cropping to facilitate analysis and presentation. For example, original 5‐megapixel images (2776 × 2074 pixels) can be partitioned into non‐overlapping regions of standardised dimensions, such as 1200 × 750 pixels, to focus on areas of interest while excluding peripheral or non‐informative regions. These cropped segments serve as reference images for subsequent evaluation and explanation (Figure 16) (Mantiuk et al. 2005).

**FIGURE 17:**
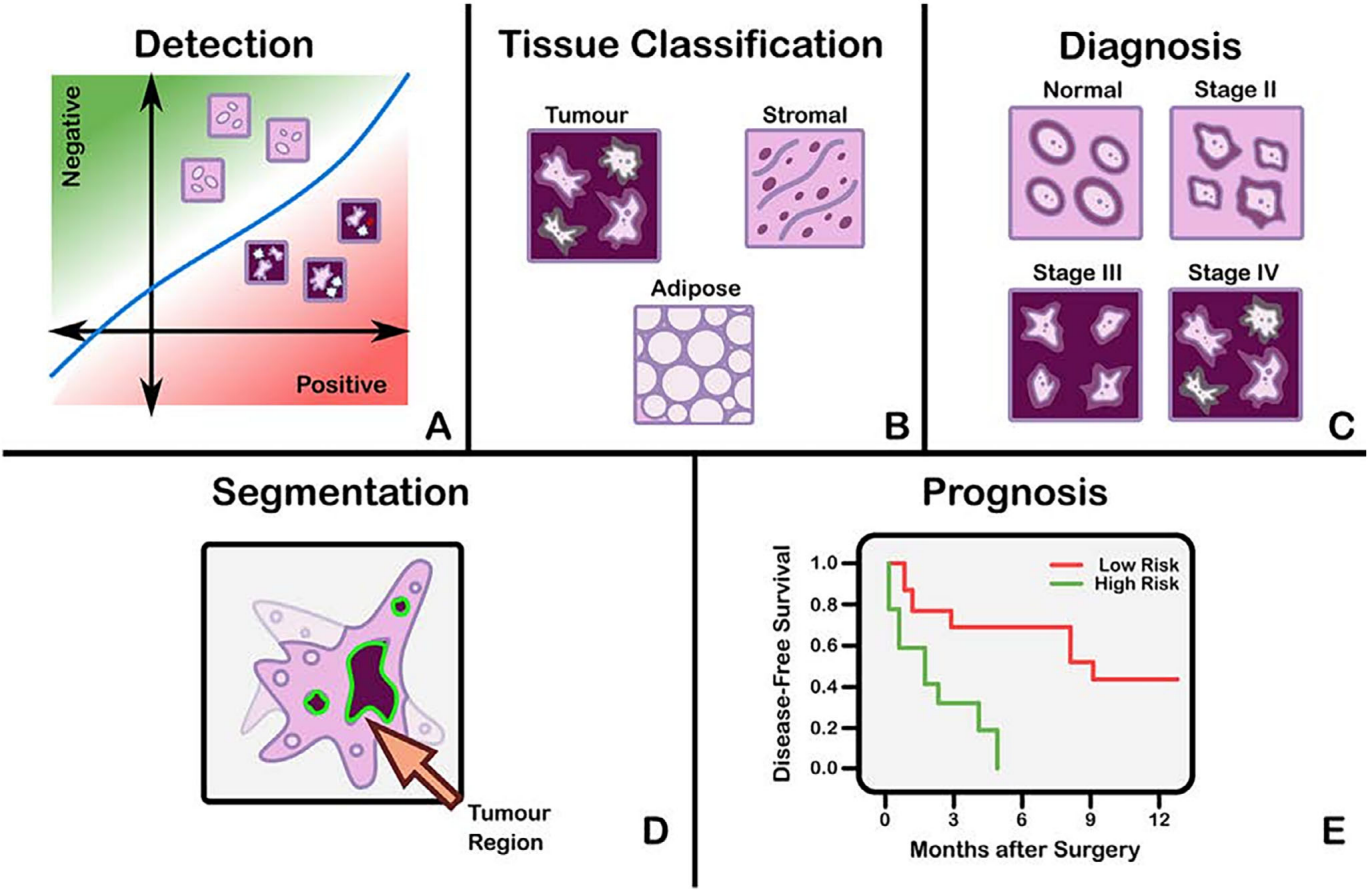
The categorisation of diagnostic tasks in computational pathology: (a) detection: a common detection task involves distinguishing positive from negative classes, such as differentiating malignant from benign findings. (b) *Tissue subtype classification*: a classification task used to identify tissue types, including tumour tissue, stroma and adipose tissue. (c) *Disease diagnosis*: a typical diagnostic task such as cancer staging. (d) *Segmentation*: tumour segmentation performed on whole‐slide images (WSIs). (e) *Prognosis tasks*: illustrated by a graph depicting survival rate in relation to the number of months following surgery (Hosseini et al. 2024).

Histopathological image analysis, whether based on ML, deep learning or more conventional computational approaches, typically falls into several broad categories, including segmentation, classification, detection and prediction (Figure 17) (Hosseini et al. 2024). Segmentation refers to the precise delineation of the boundaries of a specific tissue component from its surrounding structures. Commonly segmented elements include epithelia, glands, stroma, individual cells and nuclei (Kiehl 2022). Classification, by contrast, generally relies on various clustering methodologies, including supervised clustering when labelled or annotated data are available and unsupervised clustering in situations where such labels are limited. Through these approaches, images are categorised into defined groups, such as tumour versus non‐tumour, tumour subtypes or tumour grades (Coudray et al. 2018).

Moreover, segmentation represents advancement beyond classification by incorporating spatial localisation into predicted labels. In semantic segmentation, objects of interest within an image are delineated by assigning a class label to each pixel (Platiša et al. 2017). These class labels may be discrete or continuous, with the latter presenting a more challenging task. Another variant, instance segmentation, seeks to achieve both pixel‐level segmentation precision and clearly defined object (instance) boundaries. Segmentation techniques can accurately capture a range of morphological statistics and textural features, both of which are pertinent to cancer diagnosis and prognosis (Hosseini et al. 2024).

The integration of image analysis, AI and digital pathology offers significant opportunities for obtaining quantitative insights (Table 3). Researchers employ these technologies to explore the relationships between specific cellular features and disease outcomes (Farris et al. 2021). Such investigations have the potential to identify novel biomarkers, prognostic indicators and therapeutic targets, which could substantially advance the field of medical research (Kiran et al. 2023).

**TABLE 3:** Summary of conventional and modern diagnostic methods in avian pathology.

Diagnostic method	Sensitivity	Specificity	Turnaround time	Cost	References
Histopathology	Moderate	Moderate–high	1–3 days	Low–moderate	Bóna et al. (2023)
Immunohistochemistry	High	High	2–4 days	Moderate–high	Bóna et al. (2023)
Conventional PCR	Moderate to high	Moderate to high	Several hours	Moderate	Yadav et al. (2024)
Real‑time PCR (qPCR)	Very high (often > 98 %)	Very high (>98 %)	∼1–3 h	Higher	Kuchipudi et al. (2021)
RT‑LAMP	High (87.9%–99.5%, depending on assay)	High (94%–100%)	Very rapid (∼45‐60 min)	Lower than PCR	Cea‐Callejo et al. (2024)
Next‑generation sequencing	Very high	Very high	1–3 days (including analysis)	Very high	Azeem and Yoon (2025)
Digital pathology (whole‐slide imaging, WSIs)	High—depends on image quality and algorithms	High	Hours to 1 day	High	Coudray et al. (2018)
AI‐based image analysis/Machine learning	Very high	Very high	Minutes to hour	High	Coudray et al. (2018)

Abbreviations: PCR, polymerase chain reaction; WSI, whole‐slide imaging.

### 2.7 Avian Pathology Diagnostics in Ethiopia and Developed Regions

Ethiopia has made substantial advancements in avian pathology diagnostics, largely driven by the rapid growth of its poultry sector and the need to control widespread infectious diseases (Damena et al. 2016). Among these advancements, the establishment and strengthening of diagnostic laboratories has been notable, such as the National Animal Health Diagnostic and Investigation Centre (NAHDIC) in Sebeta, which serves as the national reference laboratory and coordinates with regional laboratories to form a structured diagnostic network supporting disease detection and surveillance across the country (Asfaw et al. 2021a, 2021b; Wayou et al. 2021). The development and coordination of national and regional diagnostic laboratories, as seen with NAHDIC, has strengthened disease surveillance and improved the country's capacity for timely and effective pathogen detection.

The introduction of molecular techniques represents a major step forward in Ethiopia's diagnostic capacity. Tools such as PCR and real‐time PCR are increasingly applied for the rapid and specific detection of pathogens, including avian influenza, Newcastle disease and infectious bursal disease. These methods have improved both the accuracy and timeliness of diagnosis, which is crucial for effective disease management and control strategies (Worku et al. 2022; Alemu et al. 2024). In addition to molecular diagnostics, traditional methods remain indispensable within Ethiopia's veterinary diagnostic system. Histopathology continues to provide critical insights into the cellular and tissue‐level manifestations of avian diseases, thereby complementing molecular assays and contributing to a more comprehensive understanding of disease pathogenesis (Birhan et al. 2023).

Developed regions, including North America, Western Europe and Australia, possess highly advanced, well‐funded and integrated diagnostic infrastructures. These systems are characterised by continuous innovation and strong institutional support, facilitating rapid disease detection, efficient surveillance and the effective translation of research findings into practical interventions (Yusuf et al. 2024). A key strength is the presence of comprehensive diagnostic networks, where government laboratories, university‐based diagnostic facilities and private laboratories are interconnected and supported by substantial funding. Many of these facilities are equipped with high‐containment biosafety laboratories, allowing safe handling of high‐risk avian pathogens (Xiao et al. 2025).

Molecular diagnostics in Western regions are routine, highly standardised and often automated. PCR and RT‐PCR platforms are widely employed, with multiplex assays enabling simultaneous detection of multiple pathogens. Quantitative PCR is frequently used for viral load assessment, allowing precise monitoring of infection dynamics and evaluation of disease severity (Thieulent et al. 2024).

Histopathology and IHC are highly advanced in these regions, supported by a robust system of expert veterinary pathologists and digital pathology platforms that facilitate both routine and complex diagnostic investigations (Pavone et al. 2024).

NGS is increasingly recognised as a standard diagnostic tool, with whole‐genome sequencing routinely employed for pathogen characterisation, outbreak investigation and antimicrobial resistance profiling (Tables 4 and 5) (Domrazek 2024).

**TABLE 4:** Comparison of avian pathology diagnostics in Ethiopia and developed regions.

Feature	Ethiopia	Developed regions
Basic infrastructure	Developing, with central hubs and expanding regional capacity (Canty‐Jones 2017)	Highly developed, integrated and well‐resourced diagnostic networks (FAO and IAEA 2020)
Molecular techniques	Increasingly adopted for key pathogens using PCR and RT PCR techniques (Smyth et al. 2010)	Routinely automated, multiplexed and widely implemented as a first‐line diagnostic tool (Hobbs et al. 2021)
Histopathology and IHC	Basic H&E staining is commonly used; IHC capacity is limited due to restricted antibody access (Coopersmith et al. 2025)	Advanced digital pathology, extensive IHC antibody panels and highly specialised pathologists (Chen et al. 2025)
Sequencing (NGS)	Mainly used in research or specific outbreak cases, often via external collaboration (Coopersmith et al. 2025)	Increasingly incorporated into routine diagnostics (WGS) for surveillance and outbreak response (Azeem and Yoon 2025)
Automation and throughput	Limited automation, with predominantly manual processes (Charlier et al. 2022)	Extensive automation and robotics for high‐throughput diagnostics (Velayudhan and Naikare 2022)
Data management	Emerging use of LIMS, often with manual reporting (Habte et al. 2022)	Sophisticated LIMS linked with national animal health databases (Chen et al. 2025)

Abbreviations: IHC, immunohistochemistry; LIMS, Laboratory Information Management System; NGS, next‐generation sequencing; PCR, polymerase chain reaction; RT‐PCR, real‐time polymerase chain reaction; WGS, whole genome sequencing.

**TABLE 5:** Comparative diagnostic capacity of Ethiopia and developed Regions in poultry health.

Metric	Ethiopia	Developed countries (e.g., the United States)	References
Diagnostic coverage (%)	31.6% overall; 14.5% lab‐supported	>90%	Asfaw et al. (2021a, 2021b), Nemser et al. (2025)
Number of veterinary diagnostic labs	16 (14 regional + 2 national)	45–100+	Vernooij et al. (2012), Carter and Smith (2021)
Access to labs (% reporting difficulty)	77%	5%	Ahmed et al. (2024), Nemser et al. (2025)
Molecular diagnostic capacity (%)	∼12.5% (2/16 labs)	∼90%	Wayou et al. (2021), Vudriko et al. (2021)
Average turnaround time (days)	7–14	1–3	Asfaw et al. (2021a, 2021b), Carter and Smith (2021)

## 3 Challenges in Avian Pathology

### 3.1 Emerging and Re‐Emerging Pathogens

The emergence of new diseases and the re‐emergence of recognised diseases are common occurrences in avian pathology (Scott et al. 2016). Some of these emerging diseases may have existed previously but went unrecognised due to low prevalence, mild clinical signs and lesions, lack of advanced diagnostic techniques or misdiagnosis (Brown 2008; Saif et al. 2020). Overall, these factors likely contributed to the under‐recognition of certain avian diseases, highlighting the need for enhanced surveillance and advanced diagnostic approaches.

Emerging diseases caused by novel pathogens present significant challenges in avian pathology, as they necessitate the development and implementation of rapid and accurate diagnostic methods (Orakpoghenor 2024). For instance, severe acute respiratory syndrome (SARS) is a frequently fatal infectious respiratory disease with prominent systemic symptoms. It is caused by a novel coronavirus, SARS coronavirus (SARS‐CoV), which was responsible for a global outbreak from November 2002 to July 2003. This virus is zoonotic, initially affecting wild animals, possibly bats, before spreading to other species (Zyga and Zografakis‐Sfakianakis 2011).

The development of specific molecular diagnostic assays for novel pathogens, including PCR‐based tests, has facilitated accurate diagnosis and improved understanding of the epidemiology and clinical spectrum of such diseases (Dankaona et al. 2022). This underscores the importance of rapid diagnostic development and collaborative efforts between veterinary researchers and diagnosticians in addressing emerging diseases in avian pathology (Orakpoghenor 2024).

Continuing education is also pivotal in strengthening the diagnostic capacity of veterinary pathologists. Participation in workshops, training programmes and scientific conferences on emerging poultry diseases and advanced diagnostic methods enables specialists to remain up‐to‐date with current trends and best practices in the field (Yitbarek and Dagnaw 2022; Lei et al. 2024).

### 3.2 Limited Access to Diagnostic Infrastructure

The rural location of many farms presents considerable challenges for the field application of new pathology diagnostic technologies and devices, which often require a reliable power supply and stable internet connectivity to function effectively and transmit real‐time data (Liebhart et al. 2023). When such technologies are not readily available or feasible in the field, post‐mortem examination remains a cornerstone of avian pathology, providing critical insights into the causes of morbidity and mortality observed under field conditions. Such examinations can be conducted in a timely manner if carcasses are collected without delay (Kumar et al. 2021). To ensure diagnostic reliability, they must follow a systematic approach, with all organs inspected and appropriate instruments selected according to the species. However, interpretation of lesions in birds may be hindered by advanced post‐mortem changes, often resulting from delays in carcass collection or exposure to high environmental temperatures that accelerate autolysis (Crispo 2024).

The limitations of current diagnostic tools, particularly in terms of sensitivity and specificity, present significant challenges to accurate disease diagnosis in poultry (Orakpoghenor 2024). For example, commonly used tests for avian diseases, such as serological assays for viral or bacterial infections, can produce false‐negative results, especially in early or subclinical cases (Kumar et al. 2021). As a result, infected birds may remain undetected, increasing the risk of disease spread within flocks. These limitations underscore the need for complementary diagnostic approaches with higher sensitivity to improve detection accuracy and strengthen disease control strategies (Rodrigues et al. 2017). For example, conventional diagnostic imaging techniques such as radiography demonstrate inherent weaknesses, particularly in assessing soft tissue structures and detecting subtle lesions in avian species (Low and Peh 2017). In response to such limitations, recent advancements in imaging technologies, including magnetic resonance imaging (MRI) and computed tomography (CT), have improved soft tissue visualisation and enhanced diagnostic capacity in birds (Orakpoghenor 2024).

Despite these advances, the adoption of advanced imaging modalities in avian pathology remains restricted due to their high cost and the specialised infrastructure they require (Yitbarek and Dagnaw 2022). This reality underscores the need for complementary diagnostic approaches with higher sensitivity, which can strengthen disease detection accuracy in poultry populations and facilitate more effective disease control strategies (Wang et al. 2021).

Finally, to support the successful application of advanced diagnostic tools within avian pathology practice, continuing education is essential. By equipping avian pathologists and field veterinarians with updated knowledge and skills, ongoing training enables them to adapt to new technologies and address emerging diagnostic challenges more effectively, thereby bridging the gap between technological potential and practical application (Yitbarek and Dagnaw 2022).

### 3.3 Zoonotic Risk and Biosecurity Gaps

Biosecurity practices are critical strategies aimed at preventing the introduction, spread and persistence of pathogens in livestock production (Crispo 2024). Given that pathogens can be transmitted via multiple direct and indirect routes, a comprehensive range of biosecurity measures must be implemented at the farm level (Dewulf and Van Immerseel 2019). These measures encompass routine operational practices, such as the use of farm‐specific clothing and equipment, the design of facilities that allow the segregation of zones with differing sanitary statuses, and systematic monitoring to assess the efficacy of cleaning and disinfection protocols (Delpont et al. 2023). Access of vehicles to poultry farms is strictly regulated, with entry permitted only through designated reception areas equipped with washing and disinfection stations. Similarly, all visitors are required to register upon arrival and comply with established biosecurity protocols before entering the farm (Crispo 2024).

In poultry production, biosecurity measures have received considerable and increasing attention to prevent severe and highly transmissible poultry diseases (Central Veterinary Institute et al. 2017). The poultry industry in developed regions is dominated by commercial operations, whereas in developing countries, production is largely based on village or backyard systems (Sonaiya 2007). Backyard poultry is characterised by small flocks with low levels of biosecurity, and backyard flocks represent around 80% of poultry stocks in many developing countries, often consisting of free‐ranging indigenous, unselected breeds of various ages, with multiple species mixed within the same flock (Conan et al. 2012). In backyard systems, poultry frequently interact with humans within the same household and come into close contact with wild birds and other livestock, thereby increasing opportunities for pathogen transmission (Brown 2008). These flocks are also highly exposed to vermin and predators. The absence of structured disease control strategies and the prevalence of inadequate management practices contribute to elevated baseline mortality, often attributed to predation by rodents, snakes and small carnivores, as well as infectious diseases (Abdelqader et al. 2007).

Moreover, several infectious poultry diseases are zoonotic, posing varying degrees of risk to human health. Zoonotic diseases are infections naturally transmitted between vertebrate animals and humans (Mesquita 2021). Over 60% of human pathogens are of zoonotic origin, including bacteria, viruses, fungi, protozoa and parasites (Brown 2008). In more critical cases, pathogens such as the Highly Pathogenic Avian Influenza (HPAI) A/H5N1 virus have the potential to cause fatal outcomes in both poultry and humans (Conan et al. 2012). Therefore, diagnosis is challenging in pathology because the occurrence of these zoonotic diseases, often associated with low biosecurity in poultry farms, requires the use of specialised laboratory techniques, including histopathology, IHC, digital pathology and molecular assays (Orakpoghenor 2024).

To mitigate these risks and reduce associated economic losses, poultry farms adopt biosecurity measures aimed at preventing the introduction, persistence and spread of infectious agents. These measures primarily focus on the implementation of strict isolation protocols, the regulation of human and vehicle traffic within and between farms, and the systematic application of sanitation and disinfection practices (Loth et al. 2011).

### 3.4 Antimicrobial Resistance (AMR)

Within the field of avian pathology, one of the primary drivers of diagnostic challenges is the overuse of antimicrobials during poultry production (de Mesquita Souza Saraiva et al. 2022). This practice contributes to the emergence of AMR, making the detection and management of resistant infections increasingly difficult (Panyako et al. 2022). In poultry production, a wide range of antimicrobials is commonly administered, primarily through oral routes, with the aim of preventing and treating infectious diseases as well as enhancing growth and productivity (Landoni and Albarellos 2015). However, the indiscriminate use of antimicrobials creates favourable conditions for the development of AMR in both commensal organisms and pathogenic bacteria (Hedman et al. 2020).

Beyond the emergence of resistant pathogens in poultry production, additional public health concerns arise from the presence of antimicrobial residues in poultry‐derived products, such as eggs and meat (Panyako et al. 2022). AMR arising in the poultry sector can be transmitted to humans through multiple pathways, including contaminated food or water chains, environmental pollution from poultry waste and direct contact with birds or their biological materials (Abe et al. 2016). Both the transmission of zoonotic antibiotic‐resistant bacteria and the dissemination of mobile genetic elements carrying resistance genes represent significant public health concerns (Di Francesco et al. 2021). Thereby AMR continues to pose a significant threat to both human and animal health by diminishing the effectiveness of antimicrobial agents in treating bacterial infections and increasing the risks of morbidity and mortality associated with resistant strains (Hedman et al. 2020).

**FIGURE 18:**
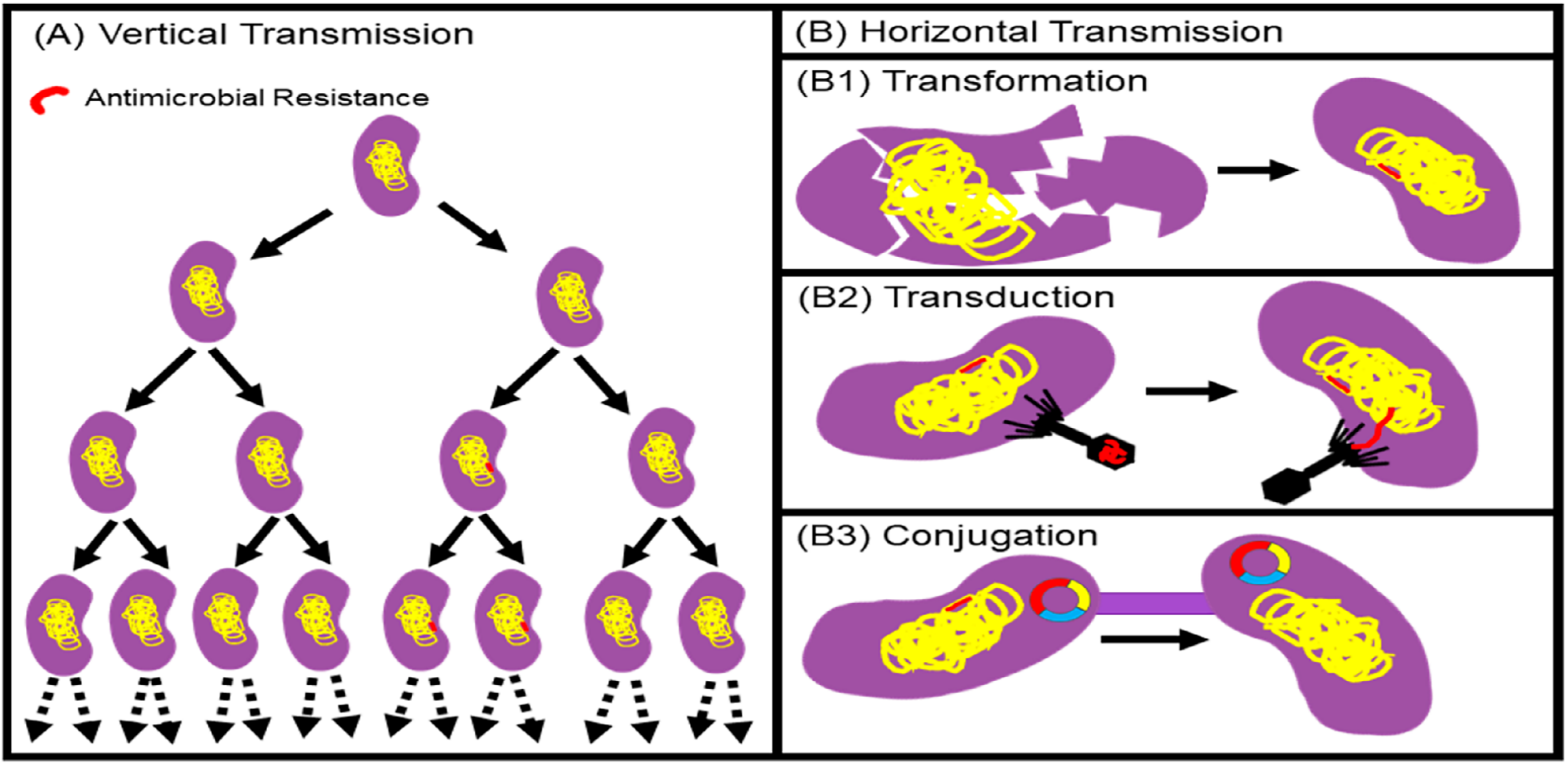
Fundamental biological pathways: primary pathways involved in the exchange of genetic information conferring antibiotic resistance, consisting of vertical transmission (a) and horizontal transmission (b) (Hedman et al. 2020).

As AMR continues to threaten both public and animal health, a clear understanding of the mechanisms driving its development is essential for monitoring its emergence and dynamics across different host populations (Chantziaras et al. 2014). Two fundamental biological pathways that facilitate the evolution and dissemination of resistance include vertical gene transfer (VGT) and horizontal gene transfer (HGT). First, AMR arises when genetic mutations that provide resistance develop within bacteria. These mutations can be transmitted from parent to daughter cells through VGT, allowing resistance traits to persist across bacterial generations (Figure 18) (Caniça et al. 2015). In the second pathway, genetic mechanisms facilitating resistance can be exchanged between bacterial species, which is also often described as HGT (Husnik and McCutcheon 2018).

Horizontal gene transfer usually manifests through the following three mechanisms: (1) transformation, defined as the exogenous DNA from environment through cell membrane, (2) transduction, defined as gene transfer from one bacterium to another through a viral medium and (3) conjugation, defined as gene transfer from a donor to a recipient cell through direct cell‐to‐cell contact mediated by plasmids (Hedman et al. 2020).

### 3.5 Inadequate Training

Insufficient training in avian pathology may result in the misdiagnosis of diseases. Without adequate knowledge of avian‐specific diagnostic algorithms, veterinarians and diagnosticians may struggle to differentiate reactive cellular changes from true neoplastic processes. Such gaps in expertise can lead to inappropriate treatment decisions or delays in the initiation of effective therapy, ultimately compromising flock health and overall disease management (Orakpoghenor 2024). Continuing education plays a crucial role in addressing these persistent challenges and ensuring that avian pathologists remain informed about advances in diagnostic techniques and disease management strategies. Participation in workshops or conferences focusing on emerging poultry diseases and novel diagnostic approaches enables veterinary pathologists to stay abreast of current trends and adopt best practices in avian health management (Wieland et al. 2021).

## 4 Future Directions

### 4.1 AI and ML

Future directions in avian pathology should emphasise the development of cost‐effective, high‐accuracy diagnostic tools, alongside the integration of AI to support rapid and precise disease diagnosis (Orakpoghenor 2024). AI facilitates image analysis, assisting pathologists in the identification of abnormalities and the diagnosis of diseases, including tumour detection, cell classification and quantitative feature assessment (Brown 2008). By standardising analytical methods and diagnostic criteria, AI reduces interpretation variability, decreases workload and improves turnaround times. Moreover, it alleviates fatigue by automating labour‐intensive and repetitive tasks, such as mitotic figure counting, cell counts and measurements of nuclear or cellular diameters (Balachandran 2025).

**FIGURE 19:**
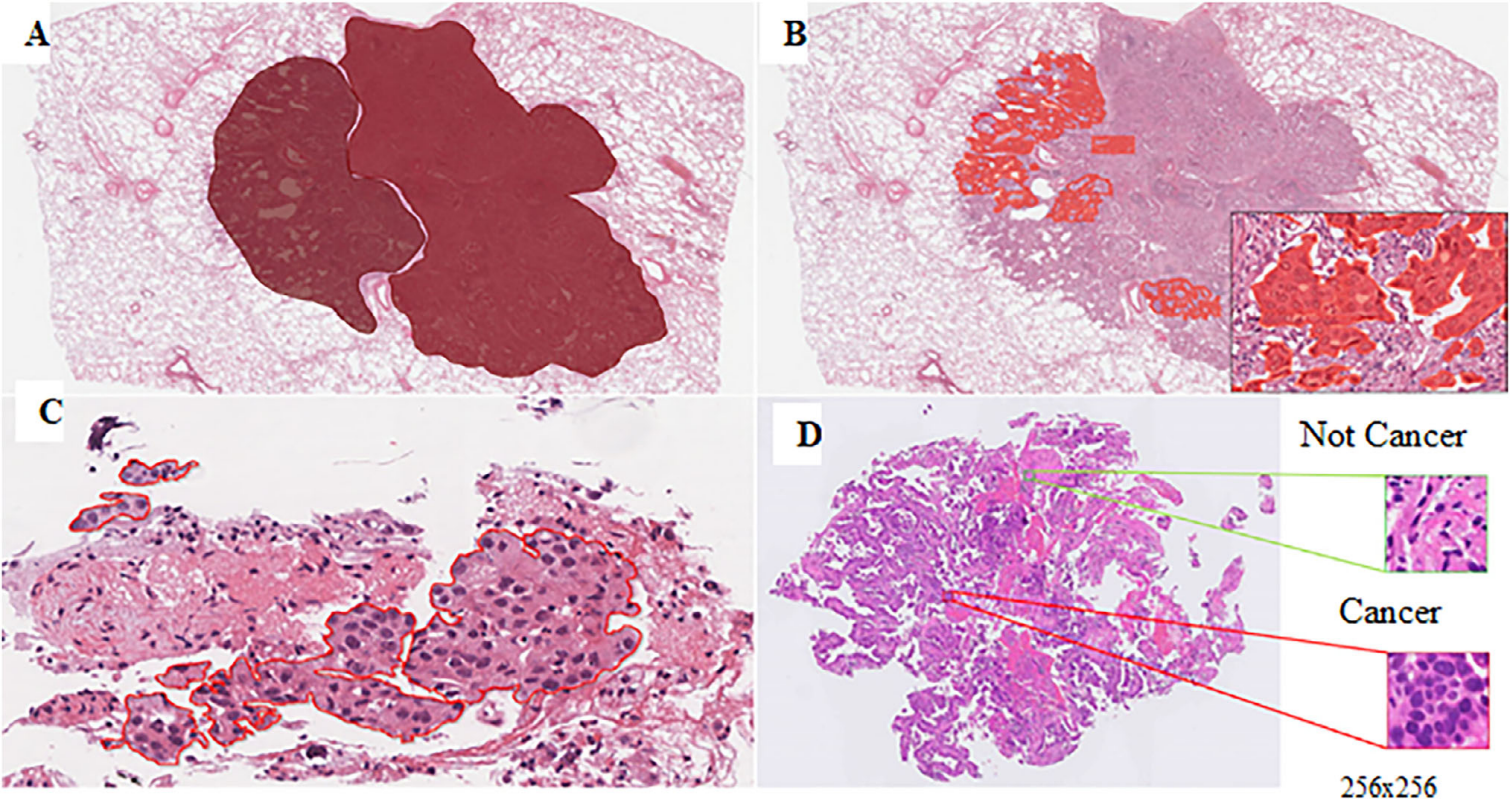
Training data for artificial intelligence: (a and b) various levels of annotation can be provided by pathologists. (c) An example of annotation with a delineated cluster of tumour cells. (d) Each WSI was cropped into small patches that were labelled either cancer or non‐cancer categories based on annotations like those in (c). Magnification: (a, b, d) whole slide; (c) 40× (Sakamoto et al. 2020).

**FIGURE 20:**
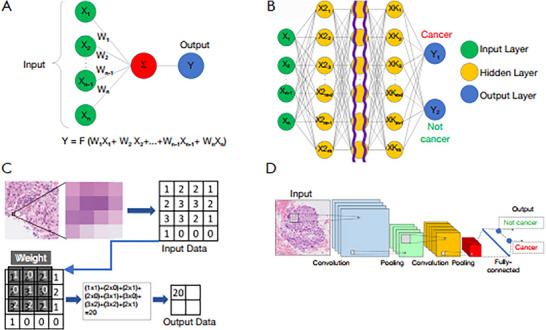
Convolutional neural network: (a) A neural network is composed of artificial neurons that assign weights (W) to individual inputs (X), such as image pixels, sum these weighted inputs and transform them into an output. (b) Layers of artificial neurons are arranged so that they feed into one another. During training, these layers progressively combine and transform the inputs to generate increasingly predictive representations of the data. As the network is repeatedly exposed to training samples, its predictions are used to update the network weights. (c) Convolution organises weights into a grid to capture the spatial structure of images, enabling the network to learn patterns characteristic of histological features. (d) Stacking multiple convolutional layers produces convolutional neural networks capable of processing complex image data effectively (Sakamoto et al. 2020).

Furthermore, the exploration of ML algorithms for poultry disease outbreak prediction and the establishment of standardised diagnostic protocols represent critical avenues for future research (Habehh and Gohel 2021). ML, a branch of AI, employs mathematical and statistical models to enable computers to learn from data and improve performance on specific tasks without the need for explicit programming or direct instruction (Balachandran 2025). The application of ML in avian pathology is primarily aimed at replicating the diagnostic strategies of pathologists, such as automating tumour grading according to conventional evaluation methods (Balachandran 2025). With the advancement of these techniques, ML has increasingly demonstrated capabilities beyond the performance limits of individual pathologists (Kiehl 2022). ML develops predictive models from data to identify underlying patterns or to perform tasks such as regression or classification (Figure 19). Within this field, deep learning represents a specialised approach in which artificial neural networks, composed of interconnected computational cells, are organised into multiple layers to emulate, in a simplified manner, the structural and functional principles of the human brain. This multi‐layered architecture enables the extraction of hierarchical features from complex datasets (Figure 20), thereby enhancing the model's capacity to learn abstract representations and achieve high levels of predictive accuracy (Sakamoto et al. 2020).

Overall, AI is already transforming the workload of avian pathologists by standardising diagnoses and reducing sources of subjectivity, including interobserver variability and differences between institutions (Fadhail 2025). In the near future, pathologists who incorporate AI into their practice are likely to be more effective than those who do not, resulting in a collaborative integration of human and machine intelligence (augmented intelligence) (Kiehl 2022).

The integration of AI and ML into biomedical diagnostics has progressed rapidly over the past decade, transforming pathology, radiology and digital signal analysis (Mohammed et al. 2020). Although applications in avian diagnostic pathology remain limited, progress in related biomedical fields offers methodological insights that can guide the development of automated, high‐precision diagnostic tools for avian diseases (Komura and Ishikawa 2018). One notable study developed a voice pathology identification system using convolutional neural networks (CNNs) combined with optimised feature‐selection methods (Mohammed et al. 2020). The system achieved high diagnostic accuracy by extracting spectro‐temporal features from vocal signals and applying deep learning for classification. The principles demonstrated, including automated feature extraction, CNN‐driven pattern recognition and end‐to‐end diagnostic pipelines, are directly applicable to avian digital pathology workflows, such as WSI segmentation and deep‐learning‐assisted lesion detection (Bertram and Klopfleisch 2017).

Similarly, the comprehensive review by Abdulmajeed et al. (2022) examined the taxonomy, diagnostic procedures and detection techniques used in voice pathology and identified challenges, including dataset imbalance, annotation burdens, feature extraction limitations and algorithm validation constraints. These issues closely mirror the diagnostic and data‐related limitations encountered in veterinary pathology, where histopathological datasets are scarce, poorly annotated and heterogeneous across staining protocols (Bertram and Klopfleisch 2017; Komura and Ishikawa 2018). The diagnostic challenges reported in human voice pathology, particularly the need for rigorous model validation, underscore the importance of robust evaluation frameworks for developing AI‐based tools in avian diagnostics.

In human digital pathology, deep learning has demonstrated exceptional performance in automated tissue segmentation, detection of neoplastic lesions and quantitative scoring of histological features (Komura and Ishikawa 2018). Models such as U‐Net, SegNet, ResNet and Deep Lab have been successfully employed to delineate tumour margins, classify inflammatory patterns and quantify cellular abnormalities (Pantanowitz et al. 2011). Similar approaches have been preliminarily applied in avian pathology, including the automated quantification of Marek's disease lesions and the detection of inclusion bodies in infectious laryngotracheitis (Bertram and Klopfleisch 2017).

Despite these advancements, the translation of AI and digital pathology technologies into avian diagnostics remains limited. Key constraints include the scarcity of large, annotated avian histopathology datasets, limited access to whole‐slide scanners, the absence of standardised formats such as DICOM‐WSI, and inadequate digital infrastructure, particularly in low‐resource regions (Afonso and Afonso 2023; Worku et al. 2022). Furthermore, the high cost of digital imaging equipment, the need for advanced computational resources and the requirement for trained personnel further restrict implementation.

Collectively, the available literature demonstrates that AI‐based diagnostic tools hold considerable promise for avian pathology; however, substantial infrastructural, technical and dataset‐related challenges must be addressed. Methodological advances in voice pathology (Abdulmajeed et al. 2022) and human digital pathology (Komura and Ishikawa 2018) provide valuable frameworks that could be adapted to improve the accuracy, speed and scalability of avian diagnostic systems.

## 5 Conclusion and Recommendations

In conclusion, advancements in avian pathology have significantly improved the detection, surveillance and management of poultry diseases through histopathology, clinical pathology, IHC, molecular diagnostics, digital pathology and AI. These technological innovations facilitate early disease detection and support outbreak prediction in poultry production. Ethiopia has achieved notable progress in avian pathology diagnostics, particularly in the application of PCR and histopathology. However, compared with Western regions with advanced molecular platforms, digital pathology and sequencing technologies, Ethiopia's system is still developing and requires further investment, infrastructure and training to meet comparable international diagnostic standards. Despite these advances, persistent challenges, such as the emergence and re‐emergence of pathogens, limited diagnostic infrastructure, biosecurity gaps and antimicrobial resistance, continue to compromise poultry health and productivity.

To address these issues, strategic recommendations include enhancing diagnostic accessibility and field‐level implementation by developing cost‐effective, rapid and farm‐applicable tools to facilitate early detection and containment of outbreaks. Strengthening biosecurity and enforcing prudent antimicrobial use are essential to limit disease spread and resistance. Investment in advanced diagnostics, including molecular techniques, digital pathology and AI‐assisted tools, will improve accuracy, efficiency and surveillance. Capacity building through targeted training for veterinary diagnosticians, avian pathologists and laboratory personnel is crucial for proficiency in emerging technologies. Research should focus on the epidemiology of emerging pathogens, the development of high‐sensitivity field diagnostics and the integration of digital pathology and AI into routine surveillance. Finally, a coordinated national strategy with centralised reporting, laboratory networking and data sharing will reinforce early warning systems and harmonise disease management.

## Author Contributions

**Gebyaw Menge Getnet**: conceptualisation, visualisation, writing – original draft, writing – review and editing. **Mengesha Ayehu Getnet**: writing – review and editing, validation and supervision. **Ayenalem Shibabaw Atenaf**: writing – review and editing.

## Funding

The authors have nothing to report.

## Ethics Statement

The authors have nothing to report.

## Consent

The authors have nothing to report.

## Conflicts of Interest

The authors declare no conflicts of interest.

## Data Availability

Data sharing not applicable to this article as no datasets were generated or analysed during the current study.
